# Within-Host Genetic Variation in Neisseria gonorrhoeae over the Course of Infection

**DOI:** 10.1128/spectrum.00313-22

**Published:** 2022-04-25

**Authors:** Jolinda de Korne-Elenbaas, Sylvia M. Bruisten, Henry J. C. de Vries, Alje P. van Dam

**Affiliations:** a Department of Infectious Diseases, Public Health Laboratory, Public Health Service of Amsterdam, Amsterdam, the Netherlands; b Amsterdam UMC, University of Amsterdam, Department of Medical Microbiology, Amsterdam Institute for Infection and Immunity (AII), location Academic Medical Center, Amsterdam, the Netherlands; c Amsterdam UMC, University of Amsterdam, Amsterdam Institute for Infection and Immunity (AII), Amsterdam, the Netherlands; d Amsterdam UMC, University of Amsterdam, Department of Dermatology, Amsterdam Institute for Infection and Immunity (AII), location Academic Medical Center, Amsterdam, the Netherlands; e Center for Sexual Health, Department of Infectious Diseases, Public Health Service Amsterdam, Amsterdam, the Netherlands; University of Pittsburgh School of Medicine

**Keywords:** *Neisseria gonorrhoeae*, SNP distance, between-host, genetic variation, recombination, transmission, within-host

## Abstract

Knowledge of within-host genetic variation informs studies on transmission dynamics. We studied within-host genetic variation in Neisseria gonorrhoeae over the course of infection and across different anatomical locations. Isolates were obtained during a clinical trial, and isolates from consecutive time points reflected persistent infections after treatment failure. We compared sequence types (STs) and recombination unfiltered- and filtered core genome single nucleotide polymorphism (SNP) distances in 65 within-host isolate pairs from the same anatomical location over time—obtained with a median interval of 7 days—and 65 isolate pairs across different anatomical locations at one time point. Isolates with different Multi-Locus Sequence Types (MLST), NG-Sequence Types for Antimicrobial Resistance (NG-STAR) and NG-Multi Antigen Sequence Types (NG-MAST) had a median of 1466 recombination filtered SNPs, whereas a median of 1 SNP was found between isolates with identical STs or a different NG-MAST only. The threshold for differentiating between strains was set at 10 recombination filtered SNPs, showing that isolates from persistent infections could have different NG-MASTs. Antibiotic pressure applied through treatment did not lead to an increase in genetic variation in specific genes or in overall extent of variation, compared to variation across anatomical locations. Instead, within-host genetic variation was proposedly driven by the host immune response, as it was concentrated in genomic regions encoding surface exposed proteins involved in host-microbe interaction. Ultimately, 15/228 (6.5%) between-host pairs contained a single strain, suggesting between-host transmission. However, patient reported data are needed to differentiate within-host persistence from between-host transmission.

**IMPORTANCE** Understanding transmission dynamics of Neisseria gonorrhoeae (*Ng*) is based on the identification of transmission events. These can be identified by assessing genetic relatedness between *Ng* isolates, expressed as core genome SNP distances. However, a SNP threshold to differentiate between strains needs to be defined, using knowledge on within- and between-host genetic variation. Here, we assessed within-host genetic variation, using a unique set of within-host *Ng* isolates from the same anatomical location over time or across different anatomical locations at one time point. The insights in genetic variation that occurred during the infection period contribute to the understanding of infection dynamics. In addition, the obtained knowledge can be used for future research on transmission dynamics and development of public health interventions based on bacterial genomic data.

## INTRODUCTION

Neisseria gonorrhoeae (*Ng*) infection is one of the most common bacterial sexually transmitted infections worldwide and the global increasing prevalence causes a high burden to public health (1). Because of the development of multidrug resistance in *Ng*, limiting the spread of *Ng* infections is needed *a fortiori*. Therefore, prevention strategies as well as prompt treatment of gonorrhea are essential. However, this is complicated by asymptomatic infections that often stay unnoticed. These asymptomatic infections, mainly rectal or pharyngeal, predominantly occur in men who have sex with men (MSM) and in women, and drive ongoing transmission (2, 3).

Research on transmission dynamics informs public health interventions targeting key populations. These interventions are based on epidemiological and behavioral factors associated with transmission within these populations, such as sexual behavior (e.g., chemsex), sex work, and PrEP use (4, 5). To understand transmission dynamics, ascertainment of a transmission event between two individuals is crucial. To study *Ng* transmission, the Neisseria gonorrhoeae Multi-Antigen Sequence Typing (NG-MAST) scheme has been created, based on two hypervariable genes (6). Comparing core genomes, instead of single genes, increases the resolution for assessing genetic relatedness between isolates and for identification of putative transmission events. For *Ng*, core genome SNP distance thresholds have been suggested for variation between isolates linked by transmission (7). These thresholds can be defined by comparing genetic variation that occurs within and between hosts, with an example of within-host genetic variation being the variation observed across different anatomical locations that are infected with the same bacterial strain. Definition of a fixed threshold is hampered by high recombination rates in *Ng*, since recombination events can lead to high SNP counts between two isolates that are identical in the rest of their genomes. Recombination filtering is therefore often applied when calculating SNP distances, although this could inflate the number of closely related isolates (8).

SNP distance thresholds to distinguish between *Ng* strains can be used for a variety of applications. As mentioned, key populations for *Ng* infection are defined based on transmission networks, identified by determining SNP distances between isolates from that population (4, 5). Modern applications provide improved partner notification based on SNP distances between isolates, potentially leading to enhanced identification of partner links (9). SNP-based methods can also be used to assess whether a bacterial strain has persisted over time or is acquired through reinfection, as done for Mycobacterium tuberculosis and different *Shigella* species (10, 11). This also provides insight in the course of infection and in variable genomic regions involved in the host-microbe interaction. For the latter purposes and to assess within-host genetic variation, the current study examined a unique collection of *Ng* isolates obtained during study visits of the New Antibiotic Treatment Options for Uncomplicated Gonorrhea clinical trial (Dutch acronym: NABOGO). This trial was performed from 2017 to 2020 at the Center for Sexual Health of Amsterdam, the Netherlands, and assessed whether gentamicin, ertapenem or fosfomycin were novel treatment options for gonorrhea (12). From cases of treatment failure, within-host isolates could be obtained from consecutive time points. To verify that treatment was truly in-effective and participants had persistent infections, reinfections with a different strain were previously ruled out based on NG-MAST typing (12). In the current study, we performed more detailed and comprehensive genetic analyses using these isolates. The availability of these isolates enabled the examination of within-host genetic changes that occur over the course of a *Ng* infection. Also, isolates from the same individual from multiple anatomical locations were obtained at one visit during the NABOGO trial. We compared the genomic variation found over time and across multiple anatomical locations to assess whether the human immune response or antibiotic pressure induced genetic variation during the infection period, either across the genome or at specific genomic regions. Ultimately, we compared within- and between-host genetic variation, to determine if cases of within-host persistence and between-host transmission could be differentiated based on core genome SNP distances.

## RESULTS

### Sequence quality and isolate pairs.

WGS was performed on 203 isolates from 80 unique participants: 74 MSM, 1 heterosexual man, 3 bisexual men and 2 women. All isolates passed quality control with a mean reference genome coverage of 98.7% (range 98.02–99.2%) and mean coverage depth of 439× (range 62–1079×) (Table S1). In total, the 203 isolates formed 65 within-host locations-pairs and 65 within-host times-pairs (Table S2). For 15 participants both locations-pairs and times-pairs were available. The 65 times-pairs were obtained from 41 participants who had *Ng* isolates available from either 2 (24/41), 3 (16/41) or 4 (1/41) time points. Days between the consecutive time points ranged from 2–23 days (median 7 days). The 65 locations-pairs were obtained from 54 participants, of whom 49 participants had a single locations-pair, 5 participants had locations-pairs from 2 time points and 3 participants had isolates from 3 anatomical locations at a single time point, which constituted 2 locations-pairs per participant. Both times- and locations-pairs were mainly obtained from men (respectively, 100% and 96%) who were MSM (respectively, 93% and 91%) and no times-pairs were obtained from women. Participants with times-pairs were predominantly allocated to the fosfomycin treatment arm (73%) whereas participants with locations-pairs were evenly distributed across the treatment arms (Table 1).

**TABLE 1:** Characteristics of participants from the NABOGO clinical trial from whom times-pairs and/or locations-pairs were obtained

	Times-pairs (*n* = 65)	Locations-pairs (*n* = 65)
No. of unique participants	41	54
Characteristic		
Sex		
Male	41 (100%)	52 (96%)
Female		2 (4%)
Sexgroup		
MSM	38 (93%)	49 (91%)
Heterosexual	1 (2%)	2 (4%)
Bisexual	2 (5%)	3 (5%)
Allocated treatment arm		
Ceftriaxone		14 (26%)
Ertapenem	1 (3%)	18 (33%)
Gentamicin	10 (24%)	14 (26%)
Fosfomycin	30 (73%)	8 (15%)

### The relationship between gene-based typing, core genome-based typing and SNP distances.

**FIG 1:**
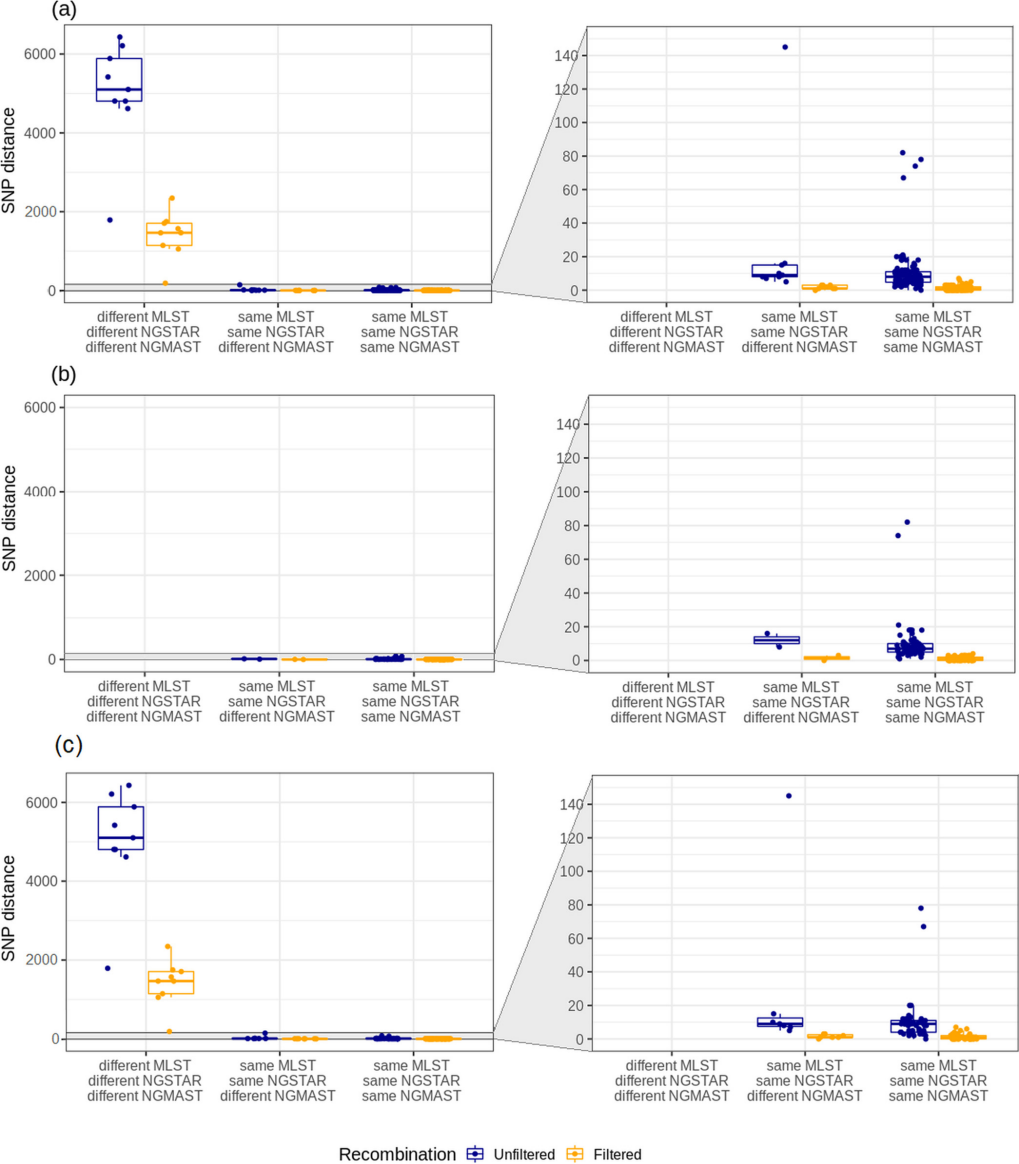
Gene-based typing results versus recombination filtered and unfiltered SNP distances between isolates in within-host pairs. MLST, NG-STAR, NG-MAST STs and SNP distances were compared between within-host paired isolates. Right panels magnify the lower values on the Y-axis. (a) SNP distances found in all within-host times-pairs. High SNP distances were found between isolates with different MLST, NG-STAR and NG-MAST STs whereas isolates that differed in NG-MAST only or isolates with identical MLST, NG-STAR and NG-MAST STs had comparable SNP distances. Zoom-in on the lower SNP distances confirmed that similar SNP distances were found between isolates with different NG-MAST compared to isolates with identical STs. Recombination filtering reduced the SNP distance to <10 SNPs for comparisons between isolates with identical STs and isolates that differed in NG-MAST only. (b) SNP distances found in within-host times-pairs. No times-pairs with different MLST, NG-STAR and NG-MAST STs were found and all times-pairs had recombination filtered SNP distances <10 SNPs. (c) SNP distances found in within-host locations-pairs. Isolates with different MLST, NG-STAR and NG-MAST STs and high SNP distances were exclusively found in locations-pairs.

Typing results and unfiltered- and recombination filtered SNPs were compared between within-host paired isolates. Pairs with identical gene-based STs (MLST, NG-STAR and NG-MAST) and pairs that differed in NG-MAST only had a median cgMLST allele distance of 0 (range 0–15), whereas pairs with different STs had a median cgMLST allele distance of 729 (range 215–949) (Fig. S1). High unfiltered SNP distances were found for pairs with different MLST, NG-STAR and NG-MAST STs, with a median of 5101 SNPs (range 1790–6432 SNPs). After recombination filtering, the SNP distances decreased to a median of 1466 SNPs (range 186–2345 SNPs) (Fig. 1a). Pairs with different NG-MAST only had much lower SNP distances that were similar to the SNP distances between pairs with identical MLST, NG-STAR and NG-MAST STs, with a median of, respectively, 9 (5–145) and 8 (range 0–82) unfiltered SNPs. After recombination filtering, the median SNP distance was 1 SNP for both groups with a maximum of 7 SNPs, showing that outliers were filtered out (Fig. 1a). Based on these results, the SNP threshold for isolates of the same strain was set at <10 recombination filtered SNPs. Isolates with <10 SNPs could have different NG-MAST STs but were still considered the same strain. When using <10 recombination filtered SNPs as reference, both MLST and NG-STAR typing methods differentiated strains in the 130 within-host pairs with 100% sensitivity and specificity, whereas NG-MAST had 93% sensitivity and 100% specificity (Table S3).

### No reinfections with distinct strains occurred in participants of the NABOGO trial.

Comparing MLST, NG-STAR and NG-MAST STs between within-host paired isolates showed identical STs in 63/65 (97%) times-pairs and 49/65 (75%) locations-pairs (Table 2). Different NG-MAST STs were found in 2/65 (3%) times-pairs and 7/65 (8%) locations-pairs, obtained from, respectively, 2 and 5 participants. The 9 isolate pairs that had identical MLST and NG-STAR STs but different NG-MAST STs all contained a single strain, based on <10 recombination filtered SNPs (Fig. 1a). Therefore, no reinfections with distinct strains occurred in participants of the NABOGO trial. However, 9/65 (14%) locations-pairs contained distinct strains, according to different MLST, NG-STAR and NG-MAST STs and high recombination filtered SNP distances (Fig. 1c). These were obtained from 8 participants, thus 8/54 (15%) participants were coinfected with distinct strains at different anatomical locations.

**TABLE 2:** Typing results of isolates in within-host times-pairs and locations-pairs

Typing results	Times-pairs (*n*= 65)	Locations-pairs (*n* = 65)
Identical MLST, NG-STAR and NG-MAST	63 (97%)	49 (75%)
Different NG-MAST, identical MLST and NG-STAR	2 (3%)	7 (11%)
Different MLST, NG-STAR and NG-MAST		9 (14%)

### Same extent of genetic variation found in times-pairs and locations-pairs.

SNP distances in within-host times-pairs and locations-pairs with a single strain were comparable, with a median of, respectively, 7 (range 1–82 SNPs) and 9 (range 0–145 SNPs) unfiltered SNPs (Fig. 1b and c). Both for within-host times-pairs and locations-pairs, the median recombination filtered SNP distance was 1 (ranges, respectively, 0–4 and 0–7 SNPs). These results show that the same extent of genetic variation arose during the infection period of at most 23 days after antibiotic treatment as well as across different anatomical locations at a single time point.

### Genetic variation was concentrated in genomic regions encoding hypervariable proteins.

**FIG 2:**
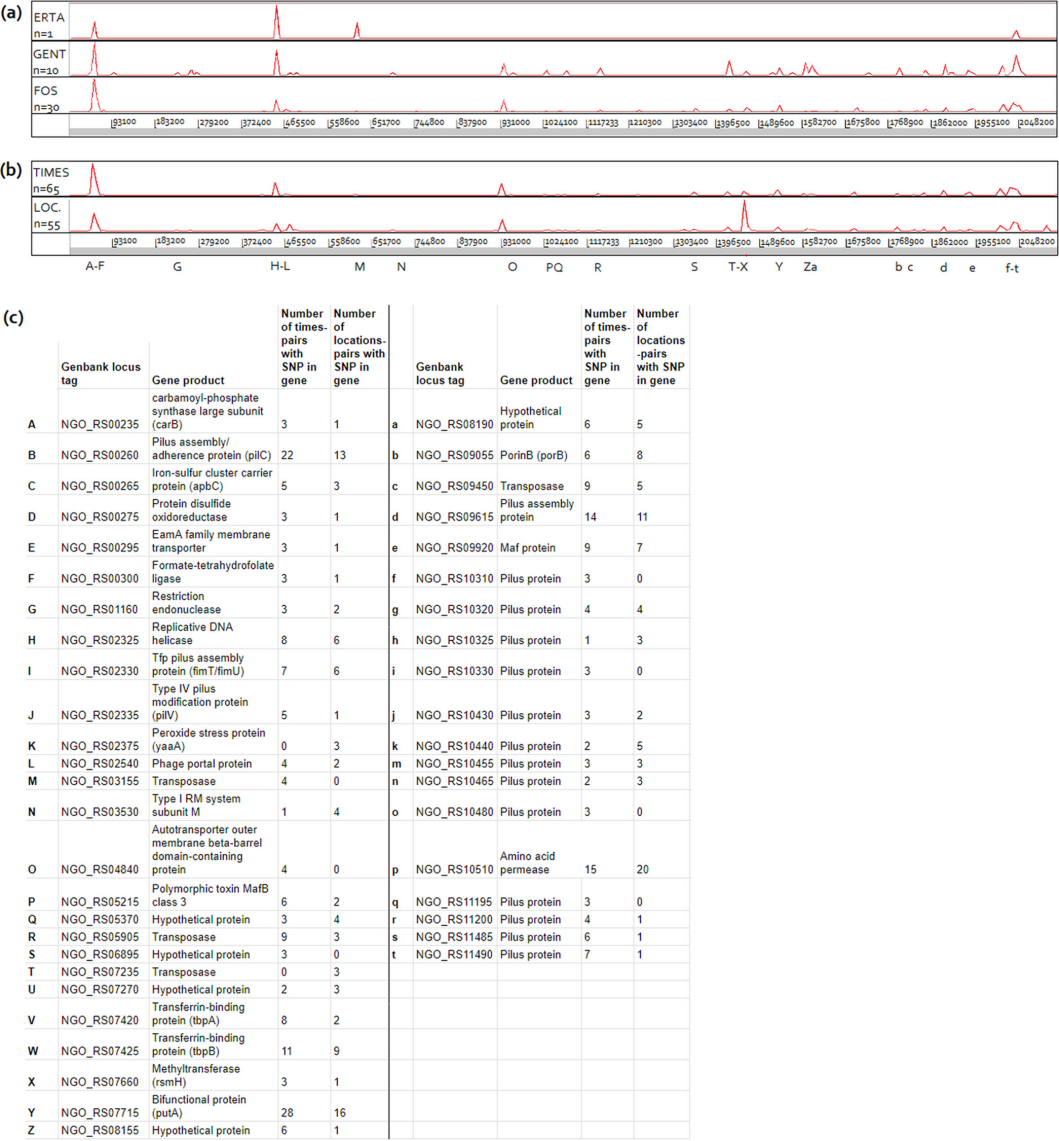
Within-host genetic variation was mainly located on regions encoding hypervariable proteins. The density of SNPs found for within-host (a) times-pairs, categorized on the allocated treatment arms, and (b) times-pairs and locations-pairs, visualized with the positions on reference genome FA1090. Letters below the graphs correspond to genes located in that region and in which multiple SNPs were found, further specified in (c). (c) Legend showing the gene ID and names that correspond to letters below graph (b), and the number of times and/or location pairs that had variation in that gene. Only genes are shown in which variation was identified in more than 2 pairs.

When visualizing genomic locations of unfiltered SNPs in within-host pairs with a single strain on reference genome FA1090, times-pairs were categorized into allocated antibiotic treatment arms. Similar genomic regions with high density SNPs were found for the different treatment arms (Fig. 2a). Also, similar variable genomic regions were identified in times-pairs and locations-pairs (Fig. 2b). Most genetic variation was found in the pilus (assembly) proteins (specifically B: NGO-RS00260 and d: NGO-RS09615), transferrin-binding proteins *tbpA* and *tbpB* (V: NGO-RS07420 and W: NGO-RS07425), bifunctional protein *putA* (Y: NGO-RS07715) and an amino acid permease (p: NGO-RS10510) and this was found in both times-pairs and locations-pairs (Fig. 2c). Remarkably, much variation was present in the genes used for NG-MAST typing (*porB* and *tbpB*) (Fig. 2c), which in some cases made up the majority of SNPs found between within-host paired isolates. In regard to the 9 within-host pairs that differed in NG-MAST STs only, 7/9 had mutations in *porB*, 1/9 had mutations in *porB* and *tbpB* and none had mutations in only *tbpB.*

### Between-host pairs with a single strain suggest cases of transmission between participants.

**FIG 3:**
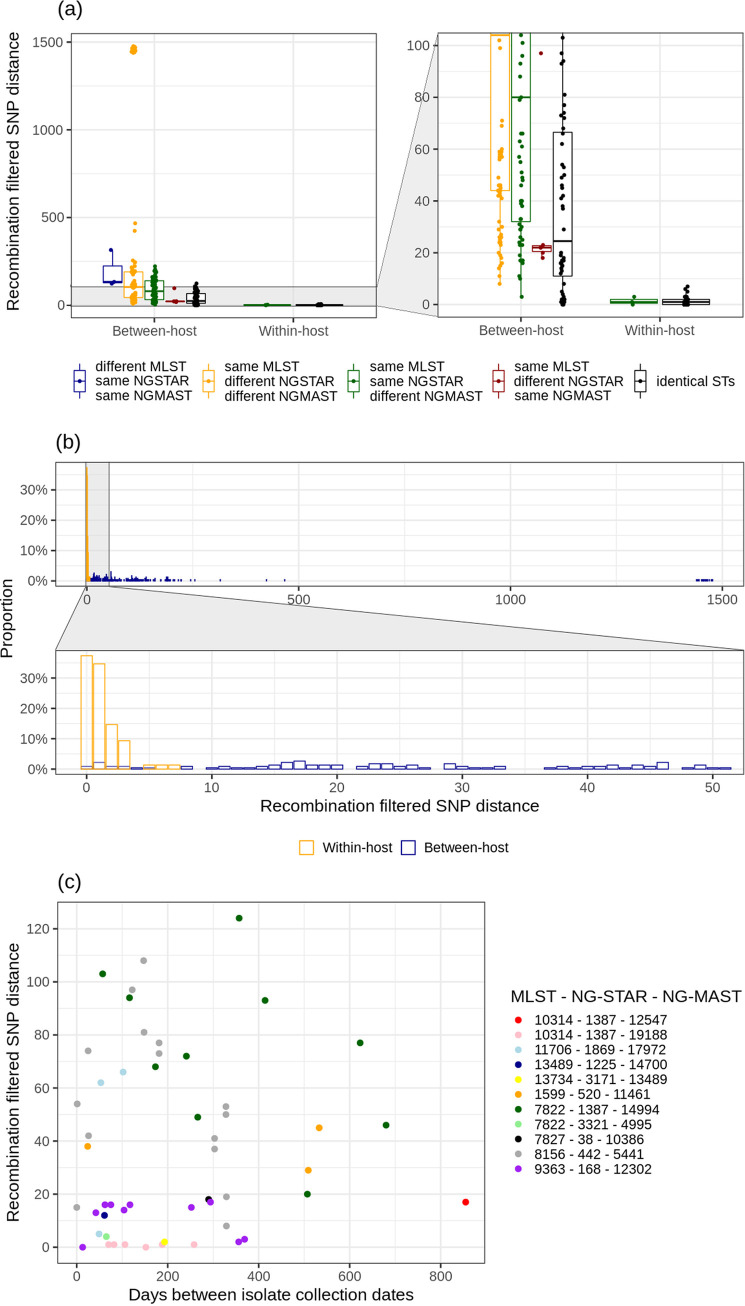
Recombination filtered SNP distances in within-host and between-host isolate pairs with identical MLST, NG-MAST and/or NG-STAR STs. (a) Recombination filtered SNP distances in within-host and between-host pairs, categorized on MLST, NG-STAR and NG-MAST typing results. Isolate pairs with different MLST, NG-STAR and NG-MAST STs were excluded. The right panel magnifies the lower values on the Y-axis. (b) Proportions of recombination filtered SNP distances found among all within-host pairs (75 pairs) and between-host pairs (228 pairs). The bottom panel magnifies the lower values on the X-axis. (c) Recombination filtered SNP distances in between-host pairs with identical MLST, NG-STAR and NG-MAST STs (52 pairs) versus the days between isolate collection dates, categorized on typing profile.

For the comparison of within- and between-host genetic variation, we used paired isolates that had at least one of the MLST, NG-STAR or NG-MAST STs identical. Seventy-five within-host pairs (only one pair per participant) and 228 between-host pairs were included (Table S4). Comparing SNP distances and STs showed broad ranges of SNP distances for between-host pairs with differences in any ST, but also between-host pairs with identical STs had up to 124 SNPs. In contrast to our finding in within-host pairs, all but one between-host pairs with different NG-MAST had ≥10 SNPs (Fig. 3a). All 75 within-host pairs had <8 recombination filtered SNPs, with 72% of SNP distances being 0 or 1. Between-host pairs resulted in more diverse recombination filtered SNP distances, with 92% between 0 and 250 SNPs (Fig. 3b). Remarkably, 15 between-host pairs (6.5%) had <10 SNPs, meaning that these pairs shared a single strain. Therefore, differentiating between- and within-host isolates is not possible based on SNP distances alone. From the 15 between-host pairs with a single strain, 13 had identical STs and 2 differed in NG-MAST only. Comparable results were found with unfiltered- instead of recombination filtered SNP distances, albeit with higher overall unfiltered SNP distances, indicating that recombination filtering did not inflate the number of closely related isolates (Fig. S2). When using <10 recombination filtered SNPs as reference, MLST differentiated between strains with 100% sensitivity but only 1% specificity in the 303 within- and between-host pairs. NG-STAR showed 99% sensitivity and 43% specificity and NG-MAST 94% sensitivity and 79% specificity (Table S3).

The SNP distances <10 SNPs for between-host pairs suggest potential direct or indirect transmission between participants. Interestingly, between-host pairs with a single strain were obtained up to 1 year apart from each other (Fig. 3c). Moreover, these pairs mainly contained isolates belonging to the MLST-NG-STAR-NG-MAST profiles 10314-1387-19188 and 9363-168-12302 (Fig. 3c), indicating probable transmission between multiple participants within the same sexual network. Between-host paired isolates with identical STs and SNP distances ≥10 SNPs belonged mainly to the MLST-NG-STAR-NG-MAST profiles 8156-442-5441 and 7822-1387-14994, which belonged to the predominant typing profiles in the study population, indicating that these strains circulated more broadly in Amsterdam and were not restricted to a single sexual network.

## DISCUSSION

We investigated within-host genetic variation in *Ng* over the course of infection, using a unique set of within-host isolates from consecutive time points. Isolates were obtained from participants with treatment failure in a randomized clinical trial comparing 4 different antibiotics for the treatment of gonorrhea (12). The genetic variation between within-host isolates over time was compared to the genetic variation between within-host isolates from different anatomical locations at a single time point. Paired isolates that differed in all gene-based STs (MLST, NG-STAR, and NG-MAST) had high core genome SNP distances, thus were defined as distinct *Ng* strains. Fifteen percent of the participants with locations-pairs had distinct strains at different anatomical locations. Similar coinfections have previously been reported in the MSM population of Amsterdam (13, 14). In contrast, a median recombination filtered SNP distance of 1 SNP was found between within-host paired isolates with identical STs or with different NG-MAST only. Since strains that are the same based on core genome SNPs could have different NG-MAST STs, this typing method alone does not identify events such as treatment failure or transmission with complete accuracy. Nevertheless, when comparing the gene-based typing methods, NG-MAST had the highest specificity for differentiating strains in between-host pairs with identical STs for at least one of the three typing methods (Table S3). This showed that NG-MAST is the most appropriate of these methods for transmission studies; however, transmission events might be incorrectly suggested to have taken place or, more rarely, be missed. This underlines the need for high-resolution methods like WGS in these studies.

Genetic variation in times-pairs could potentially be explained by the antibiotic pressure applied through treatment at T0. Due to low numbers of gentamicin- and ertapenem treatment failures, we could not identify whether specific mutations were associated with treatment arm. In addition, genes associated with gentamicin or fosfomycin resistance are not known. Instead, we assessed whether we could identify in times-pairs, compared to locations-pairs, a rise in genetic variation in specific genes or an overall rise in variation due to more general stress responses. These would enable the bacteria to faster pick up resistance genes or create resistance associated mutations (reviewed in reference 15). However, in our within-host times-pairs and locations-pairs, similar regions with high SNP densities were found (Fig. 2) as well as similar extents of genetic variation (Fig. 1b and c). This indicated that the antibiotic pressure applied during infection did not lead to the development of resistance in specific genes or to an increased extent of genetic variation. Importantly, treatment failure was neither caused by a resistant strain at the inclusion visit, since MICs were not associated with treatment failure (12). Thus, treatment failure was most likely caused by pharmacokinetic and/or dynamic reasons, e.g., suboptimal mucosal antibiotic concentrations.

Within-host genetic variation could more easily be studied in the closest relative of *Ng*, Neisseria meningitidis (*Nm*), since asymptomatic carriage of *Nm* is no indication for antibiotic treatment whereas it is for *Ng.* Several studies reported that during carriage of *Nm*, the majority of within-host genetic variation was observed in hypervariable genomic regions (16–18). We also found genetic variation mainly concentrated in hypervariable regions encoding surface exposed proteins, such as type IV pili and transferrin binding proteins. These proteins are known to play important roles in the host-microbe interaction (19, 20). This implies that both in *Nm* and *Ng*, a majority of within-host genetic variation leads to antigenic variation, driven by the host immune response.

Since *Ng* is a pathogen with high recombination rates, it is important to examine the effect of masking recombination when studying within-host variation and between-host transmission dynamics. De Silva et al. used recombination filtered SNP distances and mentioned that recombination filtering was essential for identification of transmission clusters (7). Kwong et al. used recombination filtered SNP distances to determine genetic relatedness of isolates from men in partnerships and showed that filtering enabled identification of related isolates in partners, whereas unfiltered SNP distances would have distinguished them (21). Kong et al. showed that recombination filtered SNP distances not only correctly identified partner links, but in addition identified links between individuals that were not identified as partners, most likely through indirect transmission or anonymous contacts. This technique could therefore be useful when identifying transmission networks and when implementing public health outreach interventions (9). In contrast, Williamson et al. used unfiltered SNP distances to identify potential transmission events, to use a more stringent similarity threshold in a geographically and temporally limited data set. Distinct transmission clusters could be identified using this method (5). Recombination filtering was discouraged for studies on transmission dynamics of pathogens with high interspecies variability, such as Escherichia coli. For these pathogens, calculating SNPs after filtering recombination led to loss of resolution and spurious clustering of isolates (8). Altogether, these studies show that whether or not to filter out recombination depends on the research question and should therefore be carefully considered and evaluated. The results presented in this study supported the use of recombination filtered SNP distances when studying within-host genetic diversity, since unfiltered SNP distances include high-density SNP regions between paired isolates. This high SNP density was caused by single recombination events which led to high unfiltered SNP distance between these isolates (Fig. 2).

In this study, only SNP distances in the core genome were considered, since all isolates were mapped to reference genome FA1090. This method was chosen to enable the comparison of SNP distance across pairs and ideally, across studies that use the same reference genome. However, when interpreting the results, it should be taken into account that this method does not capture variation in genomic regions that are not in the reference genome. Another limitation was that genomes from multiple colony picks from a single sample were not analyzed. As a result, we could not exclude that SNP differences in times-pairs arose during infection or were preexisting at T0. However, De Silva et al. previously compared multiple colony picks from a single culture and found only 1 SNP, most likely caused by sequencing errors (7). Thus, analyzing multiple colonies would probably not have provided more information. Our threshold of <10 recombination filtered SNPs for within-host pairs was in accordance with results from De Silva et al., who found recombination filtered SNP distances ≤10 SNPs between sequential isolates of 6/113 individuals. They supposed that these isolate pairs resulted from treatment delay or reinfection from the same partner. We confirmed this threshold with times-pairs of 41 individuals.

The between-host isolate pairs with recombination filtered SNP distances <10 SNPs suggested direct- or indirect transmission between participants. Since the within- and between-host SNP distances overlapped, SNP distances alone cannot differentiate between cases of within-host persistence or between-host transmission. This is supported by the studies of Kwong et al. and Kong et al., who found similar relatedness between isolates from men within partnerships, between isolates from multiple anatomical locations of a single individual and between isolates that were probably linked through indirect transmission (9, 21). This shows that patient reported data remains of utmost importance in studies on transmission dynamics and in randomized clinical trials. Since the current study used isolates obtained over time, the possibility of reinfection with the same strain from a steady partner cannot be excluded. However, participants were asked to refrain from sexual intercourse during the study period, which was also recorded. Moreover, the 7 days period between isolate collection was relatively short. For these reasons, patient reported metadata assures us that the within-host paired isolates from consecutive time points were from persistent infections rather than reinfection with the same strain.

## CONCLUSIONS

Comparing within-host *Ng* isolates, our results confirm the previously defined threshold of 10 recombination filtered SNPs to differentiate between strains. Antibiotic pressure applied through treatment did not lead to an increase in genetic variation in specific genes or in extent of variation, compared to variation across anatomical locations. Instead, within-host genetic variation was mainly driven by host immunity as it was concentrated in genomic regions encoding surface exposed proteins involved in host-microbe interaction. Recombination filtered SNP distances <10 SNPs were also found between isolates from different participants, suggesting transmission. To differentiate between within-host persistence and between-host transmission, additional patient reported data remains essential.

## MATERIALS AND METHODS

### Study participants and isolates.

*Ng* isolates were obtained during the NABOGO clinical trial, performed from 18 September 2017 to 5 June 2020 at the Center for Sexual Health of Amsterdam, the Netherlands. Center visitors who tested *Ng-*positive with Nucleic Acid Amplification Test (NAAT) were asked to participate and to refrain from sexual intercourse during the study period. At the inclusion visit, participants were treated according to random assignment to one of the treatment arms (ertapenem, gentamicin, fosfomycin or ceftriaxone). Test-of-cure diagnostics were performed 7–14 days after treatment. Participants could come back before or after the test-of-cure visit in case of persisting or worsening symptoms and escape medication with ceftriaxone was then given. Routine diagnostic tests were also performed on swabs obtained during these visits. More details on the study procedure have been described earlier (12).

At each study visit, anal-, pharyngeal and urethral or vaginal swabs were obtained from participants for NAAT and for phenotypic characterization using culturing. Whereas in the previous report (12) we defined treatment failure as a *Ng-*positive NAAT at the test-of-cure visit, here we could only include participants from whom *Ng* isolates from before and after treatment were available. In addition to the isolates from consecutive time points, *Ng* isolates were included from all participants who had isolates available from multiple anatomical locations at one time point. As a result, the within-host isolate pairs obtained from a single individual were derived from i) two consecutive time points from the same anatomical location or ii) different anatomical locations at the same time point, referred to as, respectively, i) times-pair or ii) locations-pair throughout the manuscript.

### WGS and quality assessment.

After phenotypic characterization, isolates were stored at −80°C. For WGS, isolates were taken from storage and cultured overnight on a chocolate blood agar plate. DNA was extracted from pure cultures in DNA/RNA shield buffer using the ZymoBIOMICS TM Magbead DNA kit (ZYMO RESEARCH). DNA sequencing libraries were prepared using the Nextera XT DNA Library Preparation kit with Integrated DNA Technologies for Illumina DNA/RNA Unique Dual Indexes (Illumina). Short-read sequencing was done using Illumina NovaSeq 6000.

Raw reads were trimmed and filtered using fastp v0.20.1 (22). Reads were mapped to reference genome FA1090 (NC_002946.2) using BWA-MEM2 v2.2.1 to calculate the percentage of bases covered and the mean coverage depth using the SAMtools package v1.9 (23, 24). Reads were assembled with Skesa v2.4.0 and assembly quality was assessed with QUAST v5.0.2 (25, 26). In case of a final assembly length of >2.1 Mb, Kraken2 v2.1.1 was used to identify contamination and to filter out reads that did not belong to *Ng* (taxid:485) (27). Filtered reads were again assembled. Isolates with >95% coverage of reference genome and with a mean coverage depth of >50× were included in the analyses.

### Typing and SNP distance determination.

Assemblies were uploaded to the pubMLST *Neisseria* database and automatically annotated, after which Multi-Locus Sequence Types (MLST), NG-Sequence Types for Antimicrobial Resistance (NG-STAR), NG-MAST v2.0 STs and core genome MLST (cgMLST) v1.0 alleles were extracted (28). When alleles were not annotated in PubMLST, sequences were manually extracted and aligned to determine similarity of alleles between paired isolates. CgMLST allele distances were determined using cgmlst-dists v0.4.0 (https://github.com/tseemann/cgmlst-dists). For variant calling, reads were mapped on reference genome FA1090 (NC_002946.2) and SNPs were identified with Snippy v4.6.0 (https://github.com/tseemann/snippy). Default settings were used: SNPs were reported with a minimum read coverage of 10×, a minimum base quality of 13 and a read concordance of 90%. A core genome alignment was created using the Snippy-core option. Recombination was filtered out using Gubbins v2.4.1 (29) and masked in the core genome alignment using the maskrc-svg script v0.5 (https://github.com/kwongj/maskrc-svg). Recombination filtered and unfiltered SNP distances between all isolates were determined using the masked- or unmasked core genome alignment with snp-dists v0.7.0 (https://github.com/tseemann/snp-dists). Snakemake v5.31.1 was used for workflow management (30).

### Comparing gene-based typing, core genome-based typing and SNP distances.

Gene-based typing results (MLST, NG-STAR, and NG-MAST STs), core genome-based allele distances (cgMLST) and recombination unfiltered and filtered SNP distances were compared between within-host paired isolates to access the discriminatory power of the different methods. Also, comparing within-host paired isolates enabled the definition of a SNP threshold to differentiate between strains. Using this threshold, within-host times-pairs with a single strain were identified as persistent infections due to treatment failure. Times-pairs or locations-pairs with distinct strains were identified as, respectively, reinfection- or coinfection with distinct strains (Table 3). These pairs were excluded from analyses on within-host genetic variation, since these are not representative for within-host genetic variation that occurs during infection.

**TABLE 3:** Definitions of events based on expected patient reported metadata and molecular data

Event	Expected patient reported data	Expected molecular data
Within-host persistence (treatment failure)	Patient reports no sexual contact between the sampling time points.	The same strain*^a^* at consecutive time points, obtained from a single individual (before and after treatment).
Within-host coinfections with distinct strains at different anatomical locations	Patient reports sexual contact with one or more sexual partners before sampling.	The same strain*^a^* at different anatomical locations of a single individual.
Reinfection with a distinct strain	Patient reports sexual contact between the sampling time points (probably with different partners).	Distinct strain^*b*^ at consecutive time points, obtained from a single individual.
Reinfection with the same strain	Patient reports sexual contact between sampling time points (probably with the same partner).	The same strain*^a^* at consecutive time points, obtained from a single individual.
Between-host transmission	Patient reports sexual contact with one or more partners before sampling.	A single strain*^a^* obtained from patient and partner.

a Same strain: <10 SNPs between isolates.

b Distinct strains: ≥10 SNPs between isolates.

### Identification of variable genomic regions by visualizing genomic locations of unfiltered SNPs.

The genomic locations of unfiltered SNPs found in within-host pairs with a single strain were visualized to identify hot spots of mutations or recombination in the genome, potentially induced by the human immune response or antibiotic pressure. For this purpose, pairs were selected from the core genome alignment and snp-sites v2.5.1 was used to create vcf files with genomic locations of the SNPs found between isolates on reference genome FA1090 (31). The previously defined SNP threshold was used to identify pairs with distinct strains, and these were excluded from this analysis. The FA1090 reference genome was visualized in Artemis v18.1.0 together with the vcf files, to plot the SNP density across the genome (32). Also, SNP locations in the vcf files were annotated with bcftools v1.9 using the *annotate* option and the prevalence of SNPs in each gene of the FA1090 genome was extracted from the annotated vcf files (33).

### Comparing within- and between-host genetic variation.

We assessed whether SNP distances could distinguish within- and between-host isolates. Isolates from two different participants formed between-host isolate pairs. Pairs were only included in the analysis if at least one of the three STs was identical (MLST, NG-STAR or NG-MAST), since the high SNP distances between isolates that differed in all STs were irrelevant. A participant could have multiple isolate pairs when isolates were available from multiple anatomical locations and from multiple time points. To prevent sampling bias caused by multiple isolates from the same participant, each participant (within-host comparisons) or each combination of participants (between-hosts comparisons) was represented only once in this analysis. Between-host transmission was defined as a single strain in a between-host pair, using the previously defined SNP threshold (Table 3).

### Data availability.

Raw sequencing reads are available in the European Nucleotide Archive under project number PRJEB49317. Genome assemblies are available in the PubMLST Neisseria database (https://pubmlst.org/organisms/neisseria-spp). Individual ENA accession numbers, PubMLST IDs and associated metadata can be found in Table S1. The bioinformatic pipeline used in this study is available at Github (https://github.com/jolindadekorne/Within-host-genetic-variation-in-Neisseria-gonorrhoeae).
