# Supplementary material for: Within-Host Genetic Variation in Neisseria gonorrhoeae over the Course of Infection
Source: Microbiol Spectr. 2022 Apr 25;10(3):e00313-22. doi: 10.1128/spectrum.00313-22 (PMC9241688; doi:10.1128/spectrum.00313-22)
Supplement: SUPPLEMENTAL FILE 1 — Supplemental material. Download spectrum.00313-22-s001.pdf, PDF file, 0.3 MB [file spectrum.00313-22-s001.pdf]

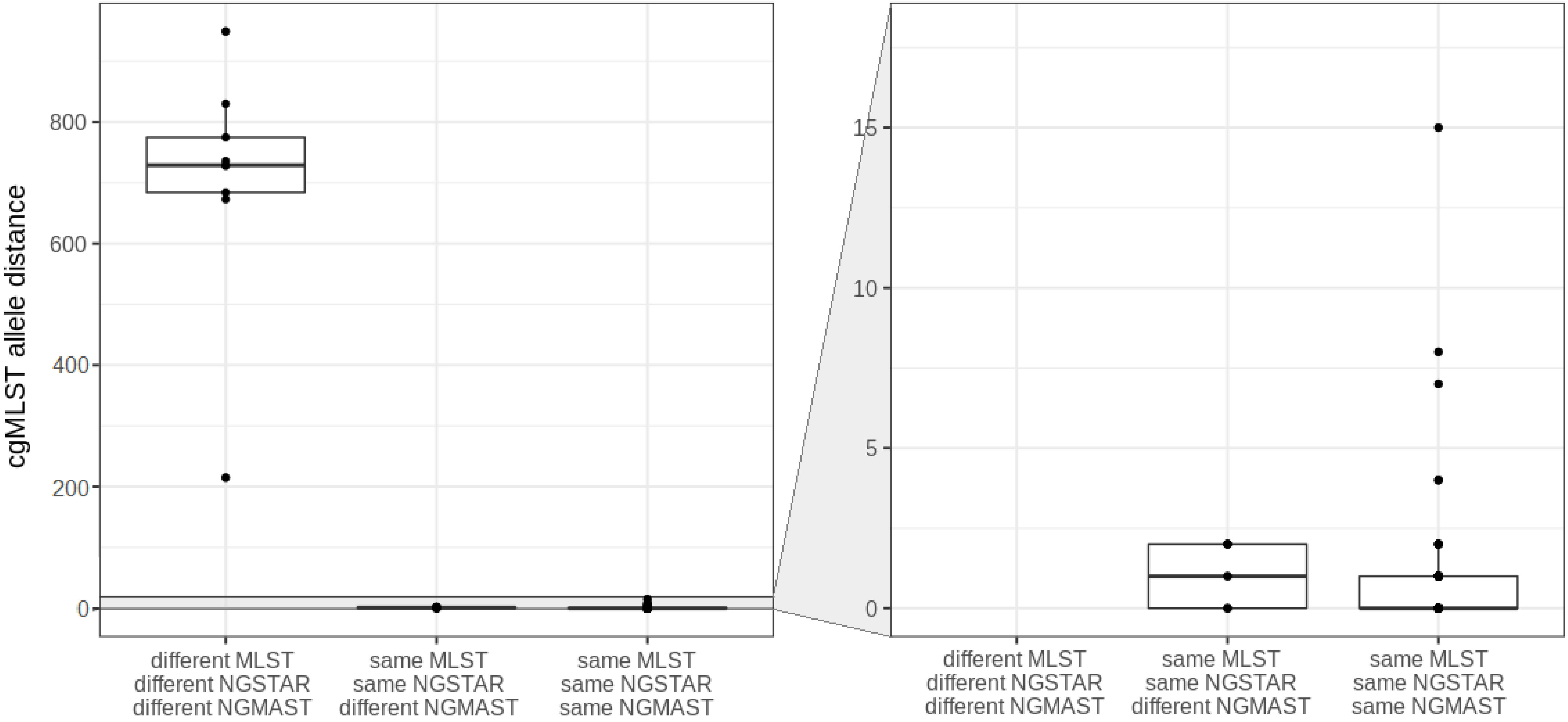

Figure S1. Gene-based typing results versus core genome MLST (cgMLST) allele distances in within-host isolate pairs. Isolates in within-host times-pairs and locations-pairs were compared by MLST, NG-STAR, NG-MAST STs and cgMLST allele distance. High cgMLST allele distances were found between isolates with different MLST, NG-STAR and NG-MAST STs whereas isolates that differed in NG-MAST only or isolates with identical MLST, NG-STAR and NG-MAST STs had comparable cgMLST allele distances. The right panel magnifies the lower values on the Y-axis, which confirms that comparable allele distances were found between isolates with different NG-MAST compared to isolates with identical STs.

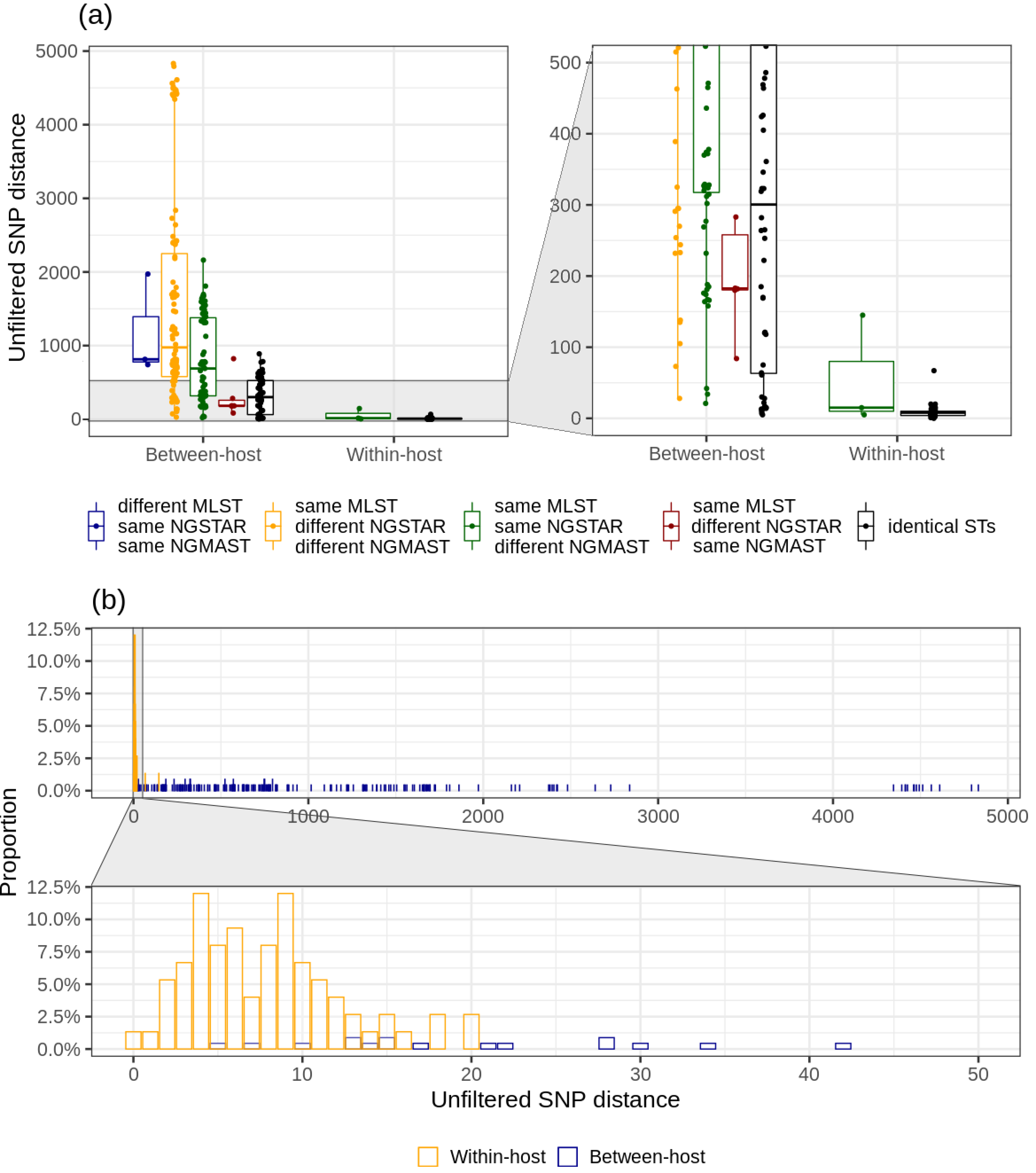

Figure S2. SNP distances in within-host and between-host isolate pairs without recombination filtering. (a) Unfiltered SNP distances in within-host and between-host isolate pairs, categorized on MLST, NG-STAR and NG-MAST typing results. Isolates pairs with different MLST, NG-STAR and NG-MAST STs were excluded. (b) Proportions of SNP distances found in all within-host pairs (75 pairs) and between-host pairs (228 pairs). Isolate pairs with different MLST, NG-STAR and NG-MAST STs were excluded.

Table S1. List of isolates included in the study with corresponding isolate- and participant metadata.

Participant number	Isolate number	ENA accession number	Year of isolation	pubMLST ID	Cove-rage of FA1090 (%)	Mean coverage depth (times)	Sex	Sex group	Allocated treatment arm	Anatomical location	Sample type	NGMAST v2.0	MLST	NG-STAR
P01	NG_AMS0386	ERS9194795	2017	99928	98.6	314	male	MSM	fosfomycin	Proctum	Times-pair	12547	10314	1387
P01	NG_AMS0388	ERS9194796	2017	99977	98.6	516	male	MSM	fosfomycin	Proctum	Times-pair	12547	10314	1387
P01	NG_AMS0382	ERS9194791	2017	100026	98.6	495	male	MSM	fosfomycin	Proctum	Times-pair	12547	10314	1387
P02	NG_AMS0384	ERS9194793	2017	99914	99	526	male	MSM	gentamycin	Pharynx	Locations-pair + times-pair	292	8122	299
P02	NG_AMS0385	ERS9194794	2017	99915	99	497	male	MSM	gentamycin	Pharynx	Times-pair	292	8122	299
P02	NG_AMS0383	ERS9194792	2017	99913	99	437	male	MSM	gentamycin	Urethra	Locations-pair	292	8122	299
P03	NG_AMS0389	ERS9194797	2017	100072	98.9	520	male	MSM	fosfomycin	Proctum	Times-pair	12302	9363	UNKN01
P03	NG_AMS0396	ERS9194804	2017	100064	98.8	482	male	MSM	fosfomycin	Proctum	Times-pair	12302	9363	UNKN01
P03	NG_AMS0399	ERS9194807	2017	100051	98.8	512	male	MSM	fosfomycin	Proctum	Times-pair	12302	9363	UNKN01
P04	NG_AMS0390	ERS9194798	2017	100047	98.8	571	male	MSM	ceftriaxon	Proctum	Locations-pair	17459	9363	301
P04	NG_AMS0391	ERS9194799	2017	100040	98.8	458	male	MSM	ceftriaxon	Pharynx	Locations-pair	17459	9363	301
P05	NG_AMS0392	ERS9194800	2017	99972	98.5	551	male	MSM	gentamycin	Proctum	Locations-pair	5441	8156	442
P05	NG_AMS0393	ERS9194801	2017	99973	98.2	139	male	MSM	gentamycin	Pharynx	Locations-pair + times-pair	5441	8156	442
P05	NG_AMS0401	ERS9194809	2017	99923	98.5	465	male	MSM	gentamycin	Pharynx	Times-pair	13784	8156	442
P06	NG_AMS0395	ERS9194803	2017	99929	98.5	546	male	MSM	ertapenem	Proctum	Locations-pair	5441	8156	442
P06	NG_AMS0394	ERS9194802	2017	99978	98.5	519	male	MSM	ertapenem	Urethra	Locations-pair	5441	8156	442
P07	NG_AMS0397	ERS9194805	2017	100011	98.9	572	male	MSM	ertapenem	Proctum	Locations-pair	5964	12093	139
P07	NG_AMS0398	ERS9194806	2017	99986	98.9	614	male	MSM	ertapenem	Pharynx	Locations-pair	5964	12093	139
P08	NG_AMS0400	ERS9194808	2017	100056	98.9	472	male	MSM	fosfomycin	Proctum	Times-pair	12302	9363	168
P08	NG_AMS0403	ERS9194811	2017	100066	98.9	599	male	MSM	fosfomycin	Proctum	Locations-pair + times-pair	12302	9363	168
P08	NG_AMS0410	ERS9194818	2017	100073	98.9	527	male	MSM	fosfomycin	Proctum	Locations-pair + times-pair	12302	9363	168
P08	NG_AMS0402	ERS9194810	2017	100065	98.9	592	male	MSM	fosfomycin	Pharynx	Locations-pair + times-pair	NEW1	9363	168
P08	NG_AMS0409	ERS9194817	2017	100067	98.9	532	male	MSM	fosfomycin	Pharynx	Locations-pair + times-pair	NEW1	9363	168
P09	NG_AMS0404	ERS9194812	2017	100009	98.6	517	male	MSM	fosfomycin	Urethra	Times-pair	14994	7822	1387
P09	NG_AMS0413	ERS9194821	2017	99959	98.5	449	male	MSM	fosfomycin	Urethra	Times-pair	14994	7822	1387
P10	NG_AMS0406	ERS9194814	2017	99904	98.5	566	male	MSM	ceftriaxon	Proctum	Locations-pair	10386	7827	38

P10	NG_AMS0405	ERS9194813	2017	99903	98.6	505	male	MSM	ceftriaxon	Urethra	Locations-pair	10386	7827	38
P10	NG_AMS0407	ERS9194815	2017	100007	98.6	430	male	MSM	ceftriaxon	Pharynx	Locations-pair	16020	10314	UNKN02
P11	NG_AMS0408	ERS9194816	2017	100068	98.9	458	male	MSM	fosfomycin	Urethra	Times-pair	12302	9363	168
P11	NG_AMS0414	ERS9194822	2017	100074	98.9	537	male	MSM	fosfomycin	Urethra	Times-pair	12302	9363	168
P12	NG_AMS0412	ERS9194820	2017	99924	98.5	532	male	bi	fosfomycin	Proctum	Locations-pair + times-pair	11461	1599	520
P12	NG_AMS0417	ERS9194825	2017	99925	98.5	510	male	bi	fosfomycin	Proctum	Locations-pair + times-pair	11461	1599	520
P12	NG_AMS0411	ERS9194819	2017	99974	98.5	561	male	bi	fosfomycin	Urethra	Locations-pair + times-pair	11461	1599	520
P12	NG_AMS0418	ERS9194826	2017	99975	98.6	564	male	bi	fosfomycin	Urethra	Locations-pair + times-pair	11461	1599	520
P13	NG_AMS0416	ERS9194824	2017	100013	98.7	496	male	MSM	ertapenem	Pharynx	Locations-pair	9918	NEW1	436
P13	NG_AMS0415	ERS9194823	2017	100012	98.7	469	male	MSM	ertapenem	Urethra	Locations-pair	9918	NEW1	436
P14	NG_AMS0419	ERS9194827	2017	99909	98.7	595	male	MSM	ceftriaxon	Pharynx	Locations-pair	15058	11971	2538
P14	NG_AMS0420	ERS9194828	2017	100016	98.6	494	male	MSM	ceftriaxon	Proctum	Locations-pair	16065	10314	1615
P15	NG_AMS0421	ERS9194829	2017	99995	98.8	435	male	MSM	fosfomycin	Proctum	Times-pair	13470	8145	3651
P15	NG_AMS0422	ERS9194830	2017	99943	98.8	583	male	MSM	fosfomycin	Proctum	Times-pair	13470	8145	3651
P16	NG_AMS0424	ERS9194832	2017	100076	98.9	493	male	MSM	ertapenem	Pharynx	Locations-pair	UNKN01	11422	2953
P16	NG_AMS0423	ERS9194831	2017	100075	98.9	483	male	MSM	ertapenem	Urethra	Locations-pair	UNKN01	11422	2953
P17	NG_AMS0425	ERS9194833	2017	99901	98.5	481	male	MSM	fosfomycin	Urethra	Times-pair	11461	1599	520
P17	NG_AMS0428	ERS9194836	2017	99895	98.5	419	male	MSM	fosfomycin	Urethra	Times-pair	11461	1599	520
P18	NG_AMS0427	ERS9194835	2017	99961	98.7	443	male	MSM	ertapenem	Proctum	Locations-pair	5624	8143	426
P18	NG_AMS0426	ERS9194834	2017	99960	98.7	474	male	MSM	ertapenem	Pharynx	Locations-pair	5624	8143	426
P19	NG_AMS0429	ERS9194837	2017	100030	98.8	493	male	MSM	fosfomycin	Proctum	Times-pair	NEW8	11428	63
P19	NG_AMS0431	ERS9194839	2017	100044	98.8	533	male	MSM	fosfomycin	Proctum	Times-pair	NEW8	11428	63
P19	NG_AMS0433	ERS9194841	2018	100032	98.8	505	male	MSM	fosfomycin	Proctum	Times-pair	NEW8	11428	63
P20	NG_AMS0430	ERS9194838	2017	100031	98.8	501	male	MSM	fosfomycin	Proctum	Times-pair	4995	7822	3321
P20	NG_AMS0432	ERS9194840	2017	100045	98.8	415	male	MSM	fosfomycin	Proctum	Times-pair	4995	7822	3321
P21	NG_AMS0435	ERS9194842	2018	100037	98.6	472	male	MSM	fosfomycin	Proctum	Times-pair	1407	1901	UNKN03
P21	NG_AMS0165_2	ERS9194781	2018	100038	98.6	461	male	MSM	fosfomycin	Proctum	Times-pair	1407	1901	UNKN03
P21	NG_AMS0166_2	ERS9194782	2018	100039	98.6	464	male	MSM	fosfomycin	Proctum	Times-pair	1407	1901	UNKN03
P22	NG_AMS0436	ERS9194843	2018	101901	98.8	532	male	MSM	gentamycin	Urethra	Locations-pair	13489	13734	3171
P22	NG_AMS0437	ERS9194844	2018	101902	98.8	502	male	MSM	gentamycin	Pharynx	Locations-pair + times-pair	12302	9363	168
P22	NG_AMS0438	ERS9194845	2018	101903	98.9	590	male	MSM	gentamycin	Pharynx	Times-pair	12302	9363	168
P23	NG_AMS0439	ERS9194846	2018	99920	98.7	452	male	MSM	fosfomycin	Urethra	Times-pair	1195	1580	182
P23	NG_AMS0440	ERS9194847	2018	99921	98.7	497	male	MSM	fosfomycin	Urethra	Times-pair	1195	1580	182
P24	NG_AMS0442	ERS9194849	2018	100062	98.9	496	male	MSM	fosfomycin	Proctum	Times-pair	12302	9363	168

P24	NG_AMS0447	ERS9194854	2018	100071	98.8	512	male	MSM	fosfomycin	Proctum	Times-pair	12302	9363	168
P25	NG_AMS0441	ERS9194848	2018	99938	98.5	454	male	MSM	fosfomycin	Urethra	Times-pair	14764	1599	520
P25	NG_AMS0443	ERS9194850	2018	99939	98.5	606	male	MSM	fosfomycin	Urethra	Times-pair	14764	1599	520
P26	NG_AMS0445	ERS9194852	2018	99996	98.8	451	male	MSM	fosfomycin	Proctum	Locations-pair	4995	7822	3321
P26	NG_AMS0444	ERS9194851	2018	99944	98.8	584	male	MSM	fosfomycin	Urethra	Locations-pair + times-pair	4995	7822	3321
P26	NG_AMS0449	ERS9194855	2018	99945	98.8	380	male	MSM	fosfomycin	Urethra	Times-pair	4995	7822	3321
P27	NG_AMS0446	ERS9194853	2018	99947	98.5	460	male	MSM	fosfomycin	Proctum	Times-pair	UNKN02	8156	442
P27	NG_AMS0451	ERS9194857	2018	99916	98.5	551	male	MSM	fosfomycin	Proctum	Locations-pair + times-pair	UNKN02	8156	442
P27	NG_AMS0452	ERS9194858	2018	99917	98.5	399	male	MSM	fosfomycin	Proctum	Locations-pair + times-pair	UNKN02	8156	442
P27	NG_AMS0450	ERS9194856	2018	100001	98.5	563	male	MSM	fosfomycin	Pharynx	Locations-pair + times-pair	UNKN03	8156	442
P27	NG_AMS0453	ERS9194859	2018	99948	98.5	392	male	MSM	fosfomycin	Pharynx	Locations-pair + times-pair	UNKN03	8156	442
P28	NG_AMS0454	ERS9194860	2018	101904	98.7	871	male	MSM	ceftriaxon	Urethra	Locations-pair	5441	8156	442
P28	NG_AMS0455	ERS9194861	2018	101905	98.9	515	male	MSM	ceftriaxon	Proctum	Locations-pair	2992	11428	63
P29	NG_AMS0456	ERS9194862	2018	99936	98.8	392	male	MSM	fosfomycin	Urethra	Times-pair	5985	12462	42
P29	NG_AMS0458	ERS9194863	2018	99937	98.8	297	male	MSM	fosfomycin	Urethra	Times-pair	5985	12462	42
P30	NG_AMS0459	ERS9194864	2018	99964	98.9	503	male	MSM	fosfomycin	Proctum	Locations-pair + times-pair	13893	13956	1295
P30	NG_AMS0462	ERS9194866	2018	99966	98.9	541	male	MSM	fosfomycin	Proctum	Locations-pair + times-pair	13893	13956	1295
P30	NG_AMS0464	ERS9194868	2018	99968	98.9	466	male	MSM	fosfomycin	Proctum	Times-pair	13893	13956	1295
P30	NG_AMS0460	ERS9194865	2018	99965	98.9	422	male	MSM	fosfomycin	Pharynx	Locations-pair + times-pair	13893	13956	1295
P30	NG_AMS0463	ERS9194867	2018	99967	98.9	474	male	MSM	fosfomycin	Pharynx	Locations-pair + times-pair	13893	13956	1295
P31	NG_AMS0465	ERS9194869	2018	100003	99.2	462	male	MSM	fosfomycin	Proctum	Times-pair	NEW2	11516	55
P31	NG_AMS0467	ERS9194871	2018	100004	99.2	534	male	MSM	fosfomycin	Proctum	Times-pair	NEW2	11516	55
P31	NG_AMS0471	ERS9194875	2018	99950	99.2	489	male	MSM	fosfomycin	Proctum	Times-pair	NEW2	11516	55
P32	NG_AMS0185_2	ERS9194784	2018	99982	98.6	570	male	MSM	gentamycin	Proctum	Locations-pair	2318	7827	38
P32	NG_AMS0184_2	ERS9194783	2018	99981	98.6	492	male	MSM	gentamycin	Urethra	Locations-pair	2318	7827	38
P33	NG_AMS0466	ERS9194870	2018	99898	98.6	552	male	hetero	fosfomycin	Urethra	Times-pair	2678	8135	729
P33	NG_AMS0468	ERS9194872	2018	99899	98.6	540	male	hetero	fosfomycin	Urethra	Times-pair	2678	8135	729
P34	NG_AMS0470	ERS9194874	2018	101907	98.6	532	male	MSM	ertapenem	Proctum	Locations-pair	19178	10314	2730
P34	NG_AMS0469	ERS9194873	2018	101906	98.6	526	male	MSM	ertapenem	Urethra	Times-pair	19178	10314	2730

P34	NG_AMS0191_2	ERS9194785	2018	101908	98.6	541	male	MSM	ertapenem	Urethra	Locations-pair + times-pair	19178	10314	2730
P35	NG_AMS0472	ERS9194876	2018	99930	98.6	471	male	MSM	fosfomycin	Urethra	Times-pair	387	11993	NEW1
P35	NG_AMS0474	ERS9194877	2018	99931	98.6	513	male	MSM	fosfomycin	Urethra	Times-pair	387	11993	NEW1
P36	NG_AMS0475	ERS9194878	2018	99983	98.8	510	male	MSM	fosfomycin	Proctum	Locations-pair + times-pair	15589	1583	3310
P36	NG_AMS0206_2	ERS9194786	2018	99932	98.8	518	male	MSM	fosfomycin	Proctum	Times-pair	15589	1583	3310
P36	NG_AMS0479	ERS9194882	2018	99985	98.9	553	male	MSM	fosfomycin	Proctum	Times-pair	15589	1583	3310
P36	NG_AMS0476	ERS9194879	2018	99984	98.8	466	male	MSM	fosfomycin	Urethra	Locations-pair	15589	1583	3310
P37	NG_AMS0477	ERS9194880	2018	99969	98.8	481	male	MSM	fosfomycin	Proctum	Times-pair	13489	13734	3171
P37	NG_AMS0482	ERS9194885	2018	99970	98.8	434	male	MSM	fosfomycin	Proctum	Times-pair	13489	13734	3171
P37	NG_AMS0486	ERS9194889	2018	99971	98.8	536	male	MSM	fosfomycin	Proctum	Times-pair	13489	13734	3171
P38	NG_AMS0478	ERS9194881	2018	100002	98.6	491	male	MSM	fosfomycin	Proctum	Times-pair	14700	13489	1225
P38	NG_AMS0485	ERS9194888	2018	100017	98.6	426	male	MSM	fosfomycin	Proctum	Times-pair	14700	13489	1225
P38	NG_AMS0491	ERS9194893	2018	100018	98.6	467	male	MSM	fosfomycin	Proctum	Times-pair	14700	13489	1225
P39	NG_AMS0481	ERS9194884	2018	99911	98.8	440	male	MSM	fosfomycin	Proctum	Locations-pair + times-pair	15589	1583	1340
P39	NG_AMS0489	ERS9194891	2018	99912	98.9	349	male	MSM	fosfomycin	Proctum	Locations-pair + times-pair	15589	1583	1340
P39	NG_AMS0495	ERS9194897	2018	99946	98.9	309	male	MSM	fosfomycin	Proctum	Times-pair	15589	1583	1340
P39	NG_AMS0480	ERS9194883	2018	99910	98.7	400	male	MSM	fosfomycin	Pharynx	Locations-pair + times-pair	15589	1583	1340
P39	NG_AMS0490	ERS9194892	2018	99997	98.7	383	male	MSM	fosfomycin	Pharynx	Locations-pair + times-pair	15589	1583	1340
P40	NG_AMS0484	ERS9194887	2018	100008	98.5	435	male	MSM	ertapenem	Proctum	Locations-pair	14994	7822	1387
P40	NG_AMS0483	ERS9194886	2018	100021	98.6	475	male	MSM	ertapenem	Urethra	Locations-pair	14994	7822	1387
P41	NG_AMS0487	ERS9194890	2018	99962	98.2	296	male	MSM	fosfomycin	Proctum	Times-pair	18147	1599	3074
P41	NG_AMS0493	ERS9194895	2018	99922	98.2	354	male	MSM	fosfomycin	Proctum	Times-pair	18147	1599	3074
P41	NG_AMS0494	ERS9194896	2018	99963	98.2	389	male	MSM	fosfomycin	Proctum	Times-pair	18147	1599	3074
P42	NG_AMS0492	ERS9194894	2018	99991	98.7	378	male	MSM	fosfomycin	Proctum	Times-pair	5441	8156	442
P42	NG_AMS0496	ERS9194898	2018	100015	98.7	338	male	MSM	fosfomycin	Proctum	Times-pair	5441	8156	442
P42	NG_AMS0499	ERS9194901	2018	99992	98.7	356	male	MSM	fosfomycin	Proctum	Times-pair	5441	8156	442
P43	NG_AMS0497	ERS9194899	2018	100081	98.8	389	male	MSM	ertapenem	Proctum	Locations-pair	18797	9363	1039
P43	NG_AMS0498	ERS9194900	2018	100082	98.8	399	male	MSM	ertapenem	Pharynx	Locations-pair	18797	9363	1039
P44	NG_AMS0500	ERS9194902	2018	100028	98.6	376	male	MSM	fosfomycin	Proctum	Times-pair	19188	10314	1387
P44	NG_AMS0221_2	ERS9194788	2018	100010	98.6	336	male	MSM	fosfomycin	Proctum	Times-pair	19188	10314	1387
P45	NG_AMS0502	ERS9194903	2018	99957	98.7	395	male	MSM	fosfomycin	Proctum	Locations-pair + times-pair	5441	8156	442

P45	NG_AMS0504	ERS9194905	2018	99958	98.7	445	male	MSM	fosfomycin	Proctum	Locations-pair + times-pair	5441	8156	442
P45	NG_AMS0220_2	ERS9194787	2018	99955	98.6	305	male	MSM	fosfomycin	Pharynx	Locations-pair + times-pair	10386	7827	38
P45	NG_AMS0223_2	ERS9194789	2018	99956	98.6	369	male	MSM	fosfomycin	Pharynx	Locations-pair + times-pair	10386	7827	38
P46	NG_AMS0503	ERS9194904	2018	99933	98.5	370	male	MSM	gentamycin	Urethra	Times-pair	5441	8156	442
P46	NG_AMS0505	ERS9194906	2018	99934	98.5	442	male	MSM	gentamycin	Urethra	Times-pair	5441	8156	442
P46	NG_AMS0507	ERS9194908	2018	100029	98.5	379	male	MSM	gentamycin	Urethra	Times-pair	5441	8156	442
P47	NG_AMS0506	ERS9194907	2018	100052	98.8	302	male	MSM	fosfomycin	Proctum	Times-pair	19665	14399	UNKN04
P47	NG_AMS0510	ERS9194911	2018	100057	98.8	348	male	MSM	fosfomycin	Proctum	Times-pair	19665	14399	UNKN04
P48	NG_AMS0508	ERS9194909	2018	100005	98.6	365	male	MSM	ceftriaxon	Proctum	Locations-pair	14700	13489	1225
P48	NG_AMS0509	ERS9194910	2018	100020	98.6	400	male	MSM	ceftriaxon	Pharynx	Locations-pair	14700	13489	1225
P49	NG_AMS0512	ERS9194913	2018	100078	98.7	372	male	bi	ceftriaxon	Pharynx	Locations-pair	17495	9363	1964
P49	NG_AMS0511	ERS9194912	2018	100077	98.7	319	male	bi	ceftriaxon	Urethra	Locations-pair	17495	9363	1964
P50	NG_AMS0514	ERS9194915	2018	99894	98.5	279	male	MSM	ertapenem	Proctum	Locations-pair	5268	10317	178
P50	NG_AMS0513	ERS9194914	2018	99897	98.5	344	male	MSM	ertapenem	Pharynx	Locations-pair	5268	10317	178
P51	NG_AMS0516	ERS9194917	2018	100055	98.9	336	male	MSM	gentamycin	Pharynx	Times-pair	12302	9363	168
P51	NG_AMS0519	ERS9194920	2018	100063	98.8	271	male	MSM	gentamycin	Pharynx	Times-pair	12302	9363	168
P51	NG_AMS0515	ERS9194916	2018	100050	98.9	427	male	MSM	gentamycin	Urethra	Times-pair	12302	9363	168
P52	NG_AMS0518	ERS9194919	2018	99980	98.6	331	male	MSM	ertapenem	Pharynx	Locations-pair	12547	7822	1387
P52	NG_AMS0517	ERS9194918	2018	100027	98.6	397	male	MSM	ertapenem	Urethra	Locations-pair	12547	7822	1387
P53	NG_AMS0521	ERS9194922	2018	100006	98.6	381	male	MSM	ceftriaxon	Proctum	Locations-pair	19188	10314	1387
P53	NG_AMS0520	ERS9194921	2018	99952	98.6	394	male	MSM	ceftriaxon	Pharynx	Locations-pair	19188	10314	1387
P54	NG_AMS0522	ERS9194923	2018	99918	98.5	382	male	bi	gentamycin	Proctum	Locations-pair	13484	1599	2152
P54	NG_AMS0245_2	ERS9194790	2018	99951	98.5	330	male	bi	gentamycin	Pharynx	Locations-pair + times-pair	13484	1599	2152
P54	NG_AMS0523	ERS9194924	2018	99902	98.5	333	male	bi	gentamycin	Pharynx	Locations-pair + times-pair	13484	1599	2152
P54	NG_AMS0524	ERS9194925	2018	101909	98.5	472	male	bi	gentamycin	Pharynx	Locations-pair + times-pair	13484	1599	2152
P54	NG_AMS0527	ERS9194928	2019	101910	98.5	529	male	bi	gentamycin	Pharynx	Locations-pair + times-pair	13484	1599	2152
P55	NG_AMS0526	ERS9194927	2019	100061	98.9	323	male	MSM	ertapenem	Proctum	Locations-pair	3935	11422	193
P55	NG_AMS0525	ERS9194926	2019	100060	98.9	358	male	MSM	ertapenem	Urethra	Locations-pair	3935	11422	193
P56	NG_AMS0528	ERS9194929	2019	101911	98.6	589	male	MSM	gentamycin	Urethra	Times-pair	UNKN04	7827	175
P56	NG_AMS0529	ERS9194930	2019	101912	98.6	639	male	MSM	gentamycin	Urethra	Times-pair	UNKN04	7827	175
P57	NG_AMS0531	ERS9194932	2019	100042	98.9	503	male	MSM	gentamycin	Pharynx	Locations-pair	2992	9362	63
P57	NG_AMS0530	ERS9194931	2019	100023	98.9	305	male	MSM	gentamycin	Urethra	Locations-pair	2992	9362	63

P58	NG_AMS0532	ERS9194933	2019	99900	99.1	1079	female	hetero	ertapenem	Proctum	Locations-pair	860	1893	1576
P58	NG_AMS0533	ERS9194934	2019	99908	99.1	294	female	hetero	ertapenem	Vagina	Locations-pair	860	1893	1576
P59	NG_AMS0534	ERS9194935	2019	99935	99	283	male	MSM	ceftriaxon	Proctum	Locations-pair	NEW3	1583	2875
P59	NG_AMS0535	ERS9194936	2019	99987	98.5	391	male	MSM	ceftriaxon	Urethra	Locations-pair	11461	UNKN01	520
P60	NG_AMS0536	ERS9194937	2019	99940	98.3	398	male	MSM	gentamycin	Proctum	Locations-pair	16039	8156	442
P60	NG_AMS0537	ERS9194938	2019	99941	98.3	380	male	MSM	gentamycin	Urethra	Locations-pair	16039	8156	442
P61	NG_AMS0538	ERS9194939	2019	99993	98.6	356	male	MSM	ertapenem	Urethra	Locations-pair	19188	10314	1387
P61	NG_AMS0539	ERS9194940	2019	99994	98.6	385	male	MSM	ertapenem	Proctum	Locations-pair	NEW4	10314	1387
P62	NG_AMS0541	ERS9194942	2019	100034	98.4	229	male	MSM	gentamycin	Pharynx	Locations-pair	14994	7822	1387
P62	NG_AMS0540	ERS9194941	2019	100014	98.6	388	male	MSM	gentamycin	Urethra	Locations-pair	14994	7822	1387
P63	NG_AMS0543	ERS9194944	2019	99919	98.5	352	male	MSM	ertapenem	Proctum	Locations-pair	11461	1599	520
P63	NG_AMS0544	ERS9194945	2019	99953	98.5	394	male	MSM	ertapenem	Pharynx	Locations-pair	11461	1599	520
P63	NG_AMS0542	ERS9194943	2019	99905	98.5	363	male	MSM	ertapenem	Urethra	Locations-pair	11461	1599	520
P64	NG_AMS0545	ERS9194946	2019	101913	98.6	479	male	MSM	gentamycin	Urethra	Locations-pair	19188	10314	1387
P64	NG_AMS0546	ERS9194947	2019	101914	99	546	male	MSM	gentamycin	Proctum	Locations-pair	UNKN08	7363	719
P65	NG_AMS0548	ERS9194949	2019	100079	98.7	363	male	MSM	ceftriaxon	Proctum	Locations-pair	NEW5	1901	128
P65	NG_AMS0547	ERS9194948	2019	100080	98.7	372	male	MSM	ceftriaxon	Pharynx	Locations-pair	NEW5	1901	128
P66	NG_AMS0549	ERS9194950	2019	100053	98.5	330	male	MSM	ertapenem	Proctum	Locations-pair	17972	11706	1869
P66	NG_AMS0550	ERS9194951	2019	101915	98.5	365	male	MSM	ertapenem	Urethra	Locations-pair	17972	11706	1869
P67	NG_AMS0552	ERS9194953	2019	100043	98.4	373	male	MSM	ceftriaxon	Proctum	Locations-pair	16928	8156	442
P67	NG_AMS0551	ERS9194952	2019	99976	98.4	328	male	MSM	ceftriaxon	Urethra	Locations-pair	16928	8156	442
P68	NG_AMS0553	ERS9194954	2019	101916	98.6	549	male	MSM	gentamycin	Urethra	Times-pair	14994	7822	1387
P68	NG_AMS0554	ERS9194955	2019	101917	98.6	561	male	MSM	gentamycin	Urethra	Times-pair	14994	7822	1387
P69	NG_AMS0555	ERS9194956	2019	99906	98.5	324	male	MSM	gentamycin	Proctum	Locations-pair	16001	1583	NEW2
P69	NG_AMS0559	ERS9194960	2019	101918	98.5	444	male	MSM	gentamycin	Pharynx	Times-pair	16001	1583	NEW2
P69	NG_AMS0556	ERS9194957	2019	99907	98.5	323	male	MSM	gentamycin	Pharynx	Locations-pair + times-pair	UNKN05	1583	NEW2
P70	NG_AMS0557	ERS9194958	2019	99926	98.5	415	male	MSM	ertapenem	Proctum	Locations-pair	17972	11706	1869
P70	NG_AMS0558	ERS9194959	2019	99927	98.4	379	male	MSM	ertapenem	Pharynx	Locations-pair	17972	11706	1869
P71	NG_AMS0561	ERS9194962	2019	100036	98.5	392	male	MSM	ceftriaxon	Proctum	Locations-pair	17972	11706	1869
P71	NG_AMS0560	ERS9194961	2019	99942	98.4	377	male	MSM	ceftriaxon	Urethra	Locations-pair	UNKN06	11706	1869
P72	NG_AMS0562	ERS9194963	2019	100019	98.6	328	male	MSM	gentamycin	Proctum	Locations-pair	14994	7822	1387
P72	NG_AMS0563	ERS9194964	2019	100046	98.6	363	male	MSM	gentamycin	Pharynx	Locations-pair	14994	7822	1387
P73	NG_AMS0564	ERS9194965	2019	100041	98.5	370	male	MSM	ceftriaxon	Proctum	Locations-pair	19559	7822	1615
P73	NG_AMS0565	ERS9194966	2019	100022	98.5	362	male	MSM	ceftriaxon	Pharynx	Locations-pair	19559	7822	1615
P74	NG_AMS0568	ERS9194968	2019	99988	98.4	371	male	MSM	gentamycin	Proctum	Locations-pair	17972	11706	1869
P74	NG_AMS0567	ERS9194967	2019	100033	98	63	male	MSM	gentamycin	Urethra	Locations-pair	17972	11706	1869
P75	NG_AMS0569	ERS9194969	2019	101920	98.8	472	male	MSM	gentamycin	Proctum	Times-pair	19275	9363	3194

P75	NG_AMS0571	ERS9194971	2019	100084	98.9	382	male	MSM	gentamycin	Proctum	Locations-pair + times-pair	19275	9363	3194
P75	NG_AMS0572	ERS9194972	2019	100085	98.9	378	male	MSM	gentamycin	Proctum	Times-pair	19275	9363	3194
P75	NG_AMS0570	ERS9194970	2019	100083	98.8	314	male	MSM	gentamycin	Pharynx	Locations-pair	19275	9363	3194
P76	NG_AMS0574	ERS9194974	2020	100049	98.8	334	male	MSM	ceftriaxon	Proctum	Locations-pair	NEW6	9363	168
P76	NG_AMS0573	ERS9194973	2020	100048	98.8	394	male	MSM	ceftriaxon	Pharynx	Locations-pair	NEW6	9363	168
P77	NG_AMS0576	ERS9194976	2020	100054	98.4	352	male	MSM	ertapenem	Urethra	Locations-pair	NEW9	11706	1869
P77	NG_AMS0575	ERS9194975	2020	100035	98.5	361	male	MSM	ertapenem	Proctum	Locations-pair	12547	10314	1387
P78	NG_AMS0579	ERS9194979	2020	100000	98.5	376	female	hetero	gentamycin	Cervix	Locations-pair	UNKN07	8156	442
P78	NG_AMS0578	ERS9194978	2020	99999	98.5	351	female	hetero	gentamycin	Proctum	Locations-pair	UNKN07	8156	442
P78	NG_AMS0577	ERS9194977	2020	99998	98.5	310	female	hetero	gentamycin	Pharynx	Locations-pair	UNKN07	8156	442
P79	NG_AMS0581	ERS9194981	2020	100059	98.9	300	male	MSM	ceftriaxon	Proctum	Locations-pair	NEW7	9362	1660
P79	NG_AMS0580	ERS9194980	2020	100058	98.9	274	male	MSM	ceftriaxon	Urethra	Locations-pair	NEW7	9362	1660
P80	NG_AMS0583	ERS9194983	2020	100025	98.5	342	male	MSM	ertapenem	Proctum	Locations-pair	3674	8156	442
P80	NG_AMS0582	ERS9194982	2020	100024	98.5	313	male	MSM	ertapenem	Pharynx	Locations-pair	3674	8156	442

Table S2. Characteristics of all within-host isolate pairs and the differences found on the levels of sequence types, cgMLST alleles and SNPs.

Isolate1	Isolate2	Participant number	Type of pair	Time-point com-parison	MLST_iso1	MLST_iso2	NGMAST_iso1	NGMAST_iso2	NGSTAR_iso1	NGSTAR_iso2	Sequence type comparison	cgMLST_allele_dist	cgMLST_cat	Un-filtered SNP distance	Recom-bination filtered SNP distance
NG_AMS0386	NG_AMS0388	P01	Times-pair	t2t3	10314	10314	12547	12547	1387	1387	same MLST+ NGSTAR+ NGMAST	0	<15	7	1
NG_AMS0382	NG_AMS0386	P01	Times-pair	t1t2	10314	10314	12547	12547	1387	1387	same MLST+ NGSTAR+ NGMAST	0	<15	7	0
NG_AMS0383	NG_AMS0384	P02	Locations-pair		8122	8122	292	292	299	299	same MLST+ NGSTAR+ NGMAST	0	<15	20	1
NG_AMS0384	NG_AMS0385	P02	Times-pair	t1t2	8122	8122	292	292	299	299	same MLST+ NGSTAR+ NGMAST	0	<15	21	2
NG_AMS0396	NG_AMS0399	P03	Times-pair	t2t3	9363	9363	12302	12302	UNKN01	UNKN01	same MLST+ NGSTAR+ NGMAST	0	<15	6	1
NG_AMS0389	NG_AMS0396	P03	Times-pair	t1t2	9363	9363	12302	12302	UNKN01	UNKN01	same MLST+ NGSTAR+ NGMAST	0	<15	7	1
NG_AMS0390	NG_AMS0391	P04	Locations-pair		9363	9363	17459	17459	301	301	same MLST+ NGSTAR+ NGMAST	0	<15	9	1
NG_AMS0392	NG_AMS0393	P05	Locations-pair		8156	8156	5441	5441	442	442	same MLST+ NGSTAR+ NGMAST	0	<15	9	1
NG_AMS0393	NG_AMS0401	P05	Times-pair	t1t2	8156	8156	5441	13784	442	442	different NGMAST	1	<15	16	3
NG_AMS0394	NG_AMS0395	P06	Locations-pair		8156	8156	5441	5441	442	442	same MLST+ NGSTAR+ NGMAST	0	<15	9	0
NG_AMS0397	NG_AMS0398	P07	Locations-pair		12093	12093	5964	5964	139	139	same MLST+ NGSTAR+ NGMAST	0	<15	20	7
NG_AMS0402	NG_AMS0403	P08	Locations-pair		9363	9363	NEW1	12302	168	168	different NGMAST	2	<15	7	2
NG_AMS0409	NG_AMS0410	P08	Locations-pair		9363	9363	NEW1	12302	168	168	different NGMAST	2	<15	10	1
NG_AMS0403	NG_AMS0410	P08	Times-pair	t2t3	9363	9363	12302	12302	168	168	same MLST+ NGSTAR+ NGMAST	1	<15	4	2
NG_AMS0400	NG_AMS0403	P08	Times-pair	t1t2	9363	9363	12302	12302	168	168	same MLST+ NGSTAR+ NGMAST	1	<15	7	3
NG_AMS0402	NG_AMS0409	P08	Times-pair	t2t3	9363	9363	NEW1	NEW1	168	168	same MLST+ NGSTAR+ NGMAST	1	<15	9	3
NG_AMS0404	NG_AMS0413	P09	Times-pair	t1t2	7822	7822	14994	14994	1387	1387	same MLST+ NGSTAR+ NGMAST	0	<15	10	0

NG_AMS0405	NG_AMS0406	P10	Locations-pair		7827	7827	10386	10386	38	38	same MLST+ NGSTAR+ NGMAST	0	<15	2	0
NG_AMS0406	NG_AMS0407	P10	Locations-pair		7827	10314	10386	16020	38	UNKN02	different MLST+ NGSTAR+ NGMAST	728	>15	4805	1466
NG_AMS0405	NG_AMS0407	P10	Locations-pair		7827	10314	10386	16020	38	UNKN02	different MLST+ NGSTAR+ NGMAST	729	>15	4807	1464
NG_AMS0408	NG_AMS0414	P11	Times-pair	t1t2	9363	9363	12302	12302	168	168	same MLST+ NGSTAR+ NGMAST	0	<15	2	0
NG_AMS0417	NG_AMS0418	P12	Locations-pair		1599	1599	11461	11461	520	520	same MLST+ NGSTAR+ NGMAST	0	<15	4	2
NG_AMS0411	NG_AMS0412	P12	Locations-pair		1599	1599	11461	11461	520	520	same MLST+ NGSTAR+ NGMAST	0	<15	13	3
NG_AMS0412	NG_AMS0417	P12	Times-pair	t1t2	1599	1599	11461	11461	520	520	same MLST+ NGSTAR+ NGMAST	0	<15	6	1
NG_AMS0411	NG_AMS0418	P12	Times-pair	t1t2	1599	1599	11461	11461	520	520	same MLST+ NGSTAR+ NGMAST	0	<15	12	1
NG_AMS0415	NG_AMS0416	P13	Locations-pair		NEW1	NEW1	9918	9918	436	436	same MLST+ NGSTAR+ NGMAST	0	<15	4	1
NG_AMS0419	NG_AMS0420	P14	Locations-pair		11971	10314	15058	16065	2538	1615	different MLST+ NGSTAR+ NGMAST	830	>15	6432	1707
NG_AMS0421	NG_AMS0422	P15	Times-pair	t1t2	8145	8145	13470	13470	3651	3651	same MLST+ NGSTAR+ NGMAST	0	<15	18	2
NG_AMS0423	NG_AMS0424	P16	Locations-pair		11422	11422	UNKN01	UNKN01	2953	2953	same MLST+ NGSTAR+ NGMAST	0	<15	3	0
NG_AMS0425	NG_AMS0428	P17	Times-pair	t1t2	1599	1599	11461	11461	520	520	same MLST+ NGSTAR+ NGMAST	0	<15	4	1
NG_AMS0426	NG_AMS0427	P18	Locations-pair		8143	8143	5624	5624	426	426	same MLST+ NGSTAR+ NGMAST	0	<15	4	2
NG_AMS0429	NG_AMS0431	P19	Times-pair	t1t2	11428	11428	NEW8	NEW8	63	63	same MLST+ NGSTAR+ NGMAST	1	<15	4	0
NG_AMS0431	NG_AMS0433	P19	Times-pair	t2t3	11428	11428	NEW8	NEW8	63	63	same MLST+ NGSTAR+ NGMAST	2	<15	9	1
NG_AMS0430	NG_AMS0432	P20	Times-pair	t1t2	7822	7822	4995	4995	3321	3321	same MLST+ NGSTAR+ NGMAST	0	<15	6	0
NG_AMS0165	NG_AMS0166	P21	Times-pair	t2t3	1901	1901	1407	1407	UNKN03	UNKN03	same MLST+ NGSTAR+ NGMAST	1	<15	10	1
NG_AMS0165	NG_AMS0435	P21	Times-pair	t1t2	1901	1901	1407	1407	UNKN03	UNKN03	same MLST+ NGSTAR+ NGMAST	0	<15	12	2

NG_AMS0436	NG_AMS0437	P22	Locations-pair		13734	9363	13489	12302	3171	168	different MLST+ NGSTAR+ NGMAST	684	>15	5887	1054
NG_AMS0437	NG_AMS0438	P22	Times-pair	t1t2	9363	9363	12302	12302	168	168	same MLST+ NGSTAR+ NGMAST	1	<15	4	1
NG_AMS0439	NG_AMS0440	P23	Times-pair	t1t2	1580	1580	1195	1195	182	182	same MLST+ NGSTAR+ NGMAST	1	<15	13	1
NG_AMS0442	NG_AMS0447	P24	Times-pair	t1t2	9363	9363	12302	12302	168	168	same MLST+ NGSTAR+ NGMAST	1	<15	6	2
NG_AMS0441	NG_AMS0443	P25	Times-pair	t1t2	1599	1599	14764	14764	520	520	same MLST+ NGSTAR+ NGMAST	0	<15	6	0
NG_AMS0444	NG_AMS0445	P26	Locations-pair		7822	7822	4995	4995	3321	3321	same MLST+ NGSTAR+ NGMAST	1	<15	5	1
NG_AMS0444	NG_AMS0449	P26	Times-pair	t1t2	7822	7822	4995	4995	3321	3321	same MLST+ NGSTAR+ NGMAST	1	<15	10	3
NG_AMS0452	NG_AMS0453	P27	Locations-pair		8156	8156	UNKN02	UNKN03	442	442	different NGMAST	2	<15	8	3
NG_AMS0450	NG_AMS0451	P27	Locations-pair		8156	8156	UNKN03	UNKN02	442	442	different NGMAST	0	<15	9	1
NG_AMS0451	NG_AMS0452	P27	Times-pair	t2t3	8156	8156	UNKN02	UNKN02	442	442	same MLST+ NGSTAR+ NGMAST	0	<15	5	0
NG_AMS0446	NG_AMS0451	P27	Times-pair	t1t2	8156	8156	UNKN02	UNKN02	442	442	same MLST+ NGSTAR+ NGMAST	0	<15	8	3
NG_AMS0450	NG_AMS0453	P27	Times-pair	t1t2	8156	8156	UNKN03	UNKN03	442	442	same MLST+ NGSTAR+ NGMAST	1	<15	18	3
NG_AMS0454	NG_AMS0455	P28	Locations-pair		8156	11428	5441	2992	442	63	different MLST+ NGSTAR+ NGMAST	673	>15	5101	1145
NG_AMS0456	NG_AMS0458	P29	Times-pair	t1t2	12462	12462	5985	5985	42	42	same MLST+ NGSTAR+ NGMAST	0	<15	5	0
NG_AMS0462	NG_AMS0463	P30	Locations-pair		13956	13956	13893	13893	1295	1295	same MLST+ NGSTAR+ NGMAST	0	<15	8	1
NG_AMS0459	NG_AMS0460	P30	Locations-pair		13956	13956	13893	13893	1295	1295	same MLST+ NGSTAR+ NGMAST	0	<15	11	0
NG_AMS0462	NG_AMS0464	P30	Times-pair	t2t3	13956	13956	13893	13893	1295	1295	same MLST+ NGSTAR+ NGMAST	0	<15	7	3
NG_AMS0459	NG_AMS0462	P30	Times-pair	t1t2	13956	13956	13893	13893	1295	1295	same MLST+ NGSTAR+ NGMAST	0	<15	9	0
NG_AMS0460	NG_AMS0463	P30	Times-pair	t1t2	13956	13956	13893	13893	1295	1295	same MLST+ NGSTAR+ NGMAST	0	<15	11	2
NG_AMS0465	NG_AMS0467	P31	Times-pair	t1t2	11516	11516	NEW2	NEW2	55	55	same MLST+ NGSTAR+ NGMAST	0	<15	3	0

NG_AMS0467	NG_AMS0471	P31	Times-pair	t2t3	11516	11516	NEW2	NEW2	55	55	same MLST+ NGSTAR+ NGMAST	0	<15	8	0
NG_AMS0184	NG_AMS0185	P32	Locations-pair		7827	7827	2318	2318	38	38	same MLST+ NGSTAR+ NGMAST	0	<15	2	0
NG_AMS0466	NG_AMS0468	P33	Times-pair	t1t2	8135	8135	2678	2678	729	729	same MLST+ NGSTAR+ NGMAST	1	<15	5	1
NG_AMS0191	NG_AMS0470	P34	Locations-pair		10314	10314	19178	19178	2730	2730	same MLST+ NGSTAR+ NGMAST	1	<15	5	0
NG_AMS0191	NG_AMS0469	P34	Times-pair	t1t2	10314	10314	19178	19178	2730	2730	same MLST+ NGSTAR+ NGMAST	0	<15	9	0
NG_AMS0472	NG_AMS0474	P35	Times-pair	t1t2	11993	11993	387	387	NEW1	NEW1	same MLST+ NGSTAR+ NGMAST	0	<15	8	1
NG_AMS0475	NG_AMS0476	P36	Locations-pair		1583	1583	15589	15589	3310	3310	same MLST+ NGSTAR+ NGMAST	1	<15	4	1
NG_AMS0206	NG_AMS0479	P36	Times-pair	t2t3	1583	1583	15589	15589	3310	3310	same MLST+ NGSTAR+ NGMAST	0	<15	2	1
NG_AMS0206	NG_AMS0475	P36	Times-pair	t1t2	1583	1583	15589	15589	3310	3310	same MLST+ NGSTAR+ NGMAST	1	<15	4	1
NG_AMS0482	NG_AMS0486	P37	Times-pair	t2t3	13734	13734	13489	13489	3171	3171	same MLST+ NGSTAR+ NGMAST	0	<15	5	1
NG_AMS0477	NG_AMS0482	P37	Times-pair	t1t2	13734	13734	13489	13489	3171	3171	same MLST+ NGSTAR+ NGMAST	1	<15	18	2
NG_AMS0478	NG_AMS0485	P38	Times-pair	t1t2	13489	13489	14700	14700	1225	1225	same MLST+ NGSTAR+ NGMAST	0	<15	15	1
NG_AMS0485	NG_AMS0491	P38	Times-pair	t2t3	13489	13489	14700	14700	1225	1225	same MLST+ NGSTAR+ NGMAST	0	<15	18	1
NG_AMS0480	NG_AMS0481	P39	Locations-pair		1583	1583	15589	15589	1340	1340	same MLST+ NGSTAR+ NGMAST	2	<15	12	1
NG_AMS0489	NG_AMS0490	P39	Locations-pair		1583	1583	15589	15589	1340	1340	same MLST+ NGSTAR+ NGMAST	8	<15	78	4
NG_AMS0481	NG_AMS0489	P39	Times-pair	t1t2	1583	1583	15589	15589	1340	1340	same MLST+ NGSTAR+ NGMAST	1	<15	10	3
NG_AMS0489	NG_AMS0495	P39	Times-pair	t2t3	1583	1583	15589	15589	1340	1340	same MLST+ NGSTAR+ NGMAST	4	<15	74	4
NG_AMS0480	NG_AMS0490	P39	Times-pair	t1t2	1583	1583	15589	15589	1340	1340	same MLST+ NGSTAR+ NGMAST	7	<15	82	3
NG_AMS0483	NG_AMS0484	P40	Locations-pair		7822	7822	14994	14994	1387	1387	same MLST+ NGSTAR+ NGMAST	0	<15	11	1

NG_AMS0493	NG_AMS0494	P41	Times-pair	t2t3	1599	1599	18147	18147	3074	3074	same MLST+ NGSTAR+ NGMAST	0	<15	6	0
NG_AMS0487	NG_AMS0493	P41	Times-pair	t1t2	1599	1599	18147	18147	3074	3074	same MLST+ NGSTAR+ NGMAST	1	<15	8	0
NG_AMS0492	NG_AMS0496	P42	Times-pair	t1t2	8156	8156	5441	5441	442	442	same MLST+ NGSTAR+ NGMAST	0	<15	9	0
NG_AMS0496	NG_AMS0499	P42	Times-pair	t2t3	8156	8156	5441	5441	442	442	same MLST+ NGSTAR+ NGMAST	1	<15	11	0
NG_AMS0497	NG_AMS0498	P43	Locations-pair		9363	9363	18797	18797	1039	1039	same MLST+ NGSTAR+ NGMAST	1	<15	4	1
NG_AMS0221	NG_AMS0500	P44	Times-pair	t1t2	10314	10314	19188	19188	1387	1387	same MLST+ NGSTAR+ NGMAST	0	<15	5	1
NG_AMS0223	NG_AMS0502	P45	Locations-pair		7827	8156	10386	5441	38	442	different MLST+ NGSTAR+ NGMAST	736	>15	4616	1570
NG_AMS0502	NG_AMS0504	P45	Times-pair	t1t2	8156	8156	5441	5441	442	442	same MLST+ NGSTAR+ NGMAST	1	<15	4	0
NG_AMS0220	NG_AMS0223	P45	Times-pair	t1t2	7827	7827	10386	10386	38	38	same MLST+ NGSTAR+ NGMAST	1	<15	1	0
NG_AMS0503	NG_AMS0505	P46	Times-pair	t1t2	8156	8156	5441	5441	442	442	same MLST+ NGSTAR+ NGMAST	0	<15	4	0
NG_AMS0505	NG_AMS0507	P46	Times-pair	t2t3	8156	8156	5441	5441	442	442	same MLST+ NGSTAR+ NGMAST	0	<15	10	1
NG_AMS0506	NG_AMS0510	P47	Times-pair	t1t2	14399	14399	19665	19665	UNKN04	UNKN04	same MLST+ NGSTAR+ NGMAST	2	<15	6	2
NG_AMS0508	NG_AMS0509	P48	Locations-pair		13489	13489	14700	14700	1225	1225	same MLST+ NGSTAR+ NGMAST	0	<15	10	1
NG_AMS0511	NG_AMS0512	P49	Locations-pair		9363	9363	17495	17495	1964	1964	same MLST+ NGSTAR+ NGMAST	1	<15	11	3
NG_AMS0513	NG_AMS0514	P50	Locations-pair		10317	10317	5268	5268	178	178	same MLST+ NGSTAR+ NGMAST	0	<15	5	0
NG_AMS0516	NG_AMS0519	P51	Times-pair	t1t2	9363	9363	12302	12302	168	168	same MLST+ NGSTAR+ NGMAST	4	<15	6	2
NG_AMS0517	NG_AMS0518	P52	Locations-pair		7822	7822	12547	12547	1387	1387	same MLST+ NGSTAR+ NGMAST	0	<15	14	1
NG_AMS0520	NG_AMS0521	P53	Locations-pair		10314	10314	19188	19188	1387	1387	same MLST+ NGSTAR+ NGMAST	1	<15	4	0
NG_AMS0245	NG_AMS0522	P54	Locations-pair		1599	1599	13484	13484	2152	2152	same MLST+ NGSTAR+ NGMAST	1	<15	8	2

NG_AMS0524	NG_AMS0527	P54	Times-pair	t3t4	1599	1599	13484	13484	2152	2152	same MLST+ NGSTAR+ NGMAST	0	<15	4	1
NG_AMS0245	NG_AMS0523	P54	Times-pair	t1t2	1599	1599	13484	13484	2152	2152	same MLST+ NGSTAR+ NGMAST	1	<15	7	2
NG_AMS0523	NG_AMS0524	P54	Times-pair	t2t3	1599	1599	13484	13484	2152	2152	same MLST+ NGSTAR+ NGMAST	0	<15	9	1
NG_AMS0525	NG_AMS0526	P55	Locations-pair		11422	11422	3935	3935	193	193	same MLST+ NGSTAR+ NGMAST	0	<15	3	1
NG_AMS0528	NG_AMS0529	P56	Times-pair	t1t2	7827	7827	UNKN04	UNKN04	175	175	same MLST+ NGSTAR+ NGMAST	1	<15	16	2
NG_AMS0530	NG_AMS0531	P57	Locations-pair		9362	9362	2992	2992	63	63	same MLST+ NGSTAR+ NGMAST	1	<15	2	0
NG_AMS0532	NG_AMS0533	P58	Locations-pair		1893	1893	860	860	1576	1576	same MLST+ NGSTAR+ NGMAST	0	<15	12	5
NG_AMS0534	NG_AMS0535	P59	Locations-pair		1583	UNKN01	NEW3	11461	2875	520	different MLST+ NGSTAR+ NGMAST	949	>15	6211	2345
NG_AMS0536	NG_AMS0537	P60	Locations-pair		8156	8156	16039	16039	442	442	same MLST+ NGSTAR+ NGMAST	0	<15	3	1
NG_AMS0538	NG_AMS0539	P61	Locations-pair		10314	10314	19188	NEW4	1387	1387	different NGMAST	2	<15	145	3
NG_AMS0540	NG_AMS0541	P62	Locations-pair		7822	7822	14994	14994	1387	1387	same MLST+ NGSTAR+ NGMAST	0	<15	9	1
NG_AMS0542	NG_AMS0543	P63	Locations-pair		1599	1599	11461	11461	520	520	same MLST+ NGSTAR+ NGMAST	0	<15	3	2
NG_AMS0542	NG_AMS0544	P63	Locations-pair		1599	1599	11461	11461	520	520	same MLST+ NGSTAR+ NGMAST	0	<15	3	0
NG_AMS0543	NG_AMS0544	P63	Locations-pair		1599	1599	11461	11461	520	520	same MLST+ NGSTAR+ NGMAST	0	<15	5	0
NG_AMS0545	NG_AMS0546	P64	Locations-pair		10314	7363	19188	UNKN08	1387	719	different MLST+ NGSTAR+ NGMAST	775	>15	5419	1750
NG_AMS0547	NG_AMS0548	P65	Locations-pair		1901	1901	NEW5	NEW5	128	128	same MLST+ NGSTAR+ NGMAST	1	<15	9	3
NG_AMS0549	NG_AMS0550	P66	Locations-pair		11706	11706	17972	17972	1869	1869	same MLST+ NGSTAR+ NGMAST	15	<15	67	6
NG_AMS0551	NG_AMS0552	P67	Locations-pair		8156	8156	16928	16928	442	442	same MLST+ NGSTAR+ NGMAST	0	<15	12	2
NG_AMS0553	NG_AMS0554	P68	Times-pair	t1t2	7822	7822	14994	14994	1387	1387	same MLST+ NGSTAR+ NGMAST	0	<15	9	0
NG_AMS0555	NG_AMS0556	P69	Locations-pair		1583	1583	16001	UNKN05	NEW2	NEW2	different NGMAST	0	<15	5	1
NG_AMS0556	NG_AMS0559	P69	Times-pair	t1t2	1583	1583	UNKN05	16001	NEW2	NEW2	different NGMAST	1	<15	8	0

NG_AMS0557	NG_AMS0558	P70	Locations-pair		11706	11706	17972	17972	1869	1869	same MLST+ NGSTAR+ NGMAST	2	<15	8	0
NG_AMS0560	NG_AMS0561	P71	Locations-pair		11706	11706	UNKN06	17972	1869	1869	different NGMAST	0	<15	15	0
NG_AMS0562	NG_AMS0563	P72	Locations-pair		7822	7822	14994	14994	1387	1387	same MLST+ NGSTAR+ NGMAST	0	<15	6	1
NG_AMS0564	NG_AMS0565	P73	Locations-pair		7822	7822	19559	19559	1615	1615	same MLST+ NGSTAR+ NGMAST	0	<15	11	0
NG_AMS0567	NG_AMS0568	P74	Locations-pair		11706	11706	17972	17972	1869	1869	same MLST+ NGSTAR+ NGMAST	0	<15	10	3
NG_AMS0570	NG_AMS0571	P75	Locations-pair		9363	9363	19275	19275	3194	3194	same MLST+ NGSTAR+ NGMAST	1	<15	12	1
NG_AMS0571	NG_AMS0572	P75	Times-pair	t2t3	9363	9363	19275	19275	3194	3194	same MLST+ NGSTAR+ NGMAST	0	<15	4	2
NG_AMS0569	NG_AMS0571	P75	Times-pair	t1t2	9363	9363	19275	19275	3194	3194	same MLST+ NGSTAR+ NGMAST	1	<15	6	1
NG_AMS0573	NG_AMS0574	P76	Locations-pair		9363	9363	NEW6	NEW6	168	168	same MLST+ NGSTAR+ NGMAST	2	<15	8	2
NG_AMS0575	NG_AMS0576	P77	Locations-pair		10314	11706	12547	NEW9	1387	1869	different MLST+ NGSTAR+ NGMAST	215	>15	1790	186
NG_AMS0577	NG_AMS0578	P78	Locations-pair		8156	8156	UNKN07	UNKN07	442	442	same MLST+ NGSTAR+ NGMAST	1	<15	10	0
NG_AMS0577	NG_AMS0579	P78	Locations-pair		8156	8156	UNKN07	UNKN07	442	442	same MLST+ NGSTAR+ NGMAST	2	<15	10	0
NG_AMS0578	NG_AMS0579	P78	Locations-pair		8156	8156	UNKN07	UNKN07	442	442	same MLST+ NGSTAR+ NGMAST	2	<15	20	0
NG_AMS0580	NG_AMS0581	P79	Locations-pair		9362	9362	NEW7	NEW7	1660	1660	same MLST+ NGSTAR+ NGMAST	0	<15	0	0
NG_AMS0582	NG_AMS0583	P80	Locations-pair		8156	8156	3674	3674	442	442	same MLST+ NGSTAR+ NGMAST	0	<15	9	0

Table S3. Sensitivity and specificity of the gene-based typing methods MLST, NG-STAR and NG-MAST.

Within-host pairs (n=130)

**MLST**	Same ST	Different ST
<10 recombination filtered SNPs	121	0
≥10 recombination filtered SNPs	0	9
	Sensitivity	100%
	Specificity	100%

**NG-STAR**	Same ST	Different ST
<10 recombination filtered SNPs	121	0
≥10 recombination filtered SNPs	0	9
	Sensitivity	100%
	Specificity	100%

**NG-MAST**	Same ST	Different ST
<10 recombination filtered SNPs	112	9
≥10 recombination filtered SNPs	0	9
	Sensitivity	93%
	Specificity	100%

Within+ between-host pairs (n=303)

**MLST**	Same ST	Different ST
<10 recombination filtered SNPs	90	0
≥10 recombination filtered SNPs	210	3
	Sensitivity	100%
	Specificity	1%

**NG-STAR**	Same ST	Different ST
<10 recombination filtered SNPs	89	1
≥10 recombination filtered SNPs	109	104
	Sensitivity	99%
	Specificity	49%

**NG-MAST**	Same ST	Different ST
<10 recombination filtered SNPs	85	5
≥10 recombination filtered SNPs	45	168
	Sensitivity	94%
	Specificity	79%

Table S4. Characteristics of included within- and between-host isolate pairs and the differences found on the levels of sequence types, cgMLST alleles and SNPs.

Isolate1	Isolate2	Participant_isolate1	Participant_isolate2	Type of comparison	Time point com-parison	MLST_iso1	MLST_iso2	NG-MAST_iso1	NG-MAST_iso2	NG-STAR_iso1	NG-STAR_iso2	Gene based typing comparison	cgMLST_allele_dist	cgMLST_cat	Un-filtered SNP distance	Recom-bination filtered SNP distance
NG_AMS0165	NG_AMS0547	P21	P65	Between-host		1901	1901	1407	NEW5	UNKN03	128	same MLST, different NGSTAR+ NGMAST	299	>15	2640	467
NG_AMS0165	NG_AMS0166	P21	P21	Within-host	t2t3	1901	1901	1407	1407	UNKN03	UNKN03	same MLST+	1	<15	10	1
NG_AMS0184	NG_AMS0528	P32	P56	Between-host		7827	7827	2318	UNKN04	38	175	same MLST, different NGSTAR+ NGMAST	61	>15	817	42
NG_AMS0184	NG_AMS0405	P32	P10	Between-host		7827	7827	2318	10386	38	38	same MLST+ NGSTAR, different NGMAST	44	>15	689	40
NG_AMS0184	NG_AMS0220	P32	P45	Between-host		7827	7827	2318	10386	38	38	same MLST+ NGSTAR, different NGMAST	60	>15	724	49
NG_AMS0184	NG_AMS0185	P32	P32	Within-host		7827	7827	2318	2318	38	38	same MLST+	0	<15	2	0
NG_AMS0191	NG_AMS0575	P34	P77	Between-host		10314	10314	19178	12547	2730	1387	same MLST, different NGSTAR+ NGMAST	87	>15	734	56
NG_AMS0191	NG_AMS0520	P34	P53	Between-host		10314	10314	19178	19188	2730	1387	same MLST, different NGSTAR+ NGMAST	103	>15	795	58
NG_AMS0191	NG_AMS0545	P34	P64	Between-host		10314	10314	19178	19188	2730	1387	same MLST, different NGSTAR+ NGMAST	104	>15	813	58
NG_AMS0191	NG_AMS0221	P34	P44	Between-host		10314	10314	19178	19188	2730	1387	same MLST, different NGSTAR+ NGMAST	103	>15	795	59
NG_AMS0191	NG_AMS0538	P34	P61	Between-host		10314	10314	19178	19188	2730	1387	same MLST, different NGSTAR+ NGMAST	100	>15	770	59
NG_AMS0191	NG_AMS0469	P34	P34	Within-host	t1t2	10314	10314	19178	19178	2730	2730	same MLST+	0	<15	9	0
NG_AMS0206	NG_AMS0534	P36	P59	Between-host		1583	1583	15589	NEW3	3310	2875	same MLST, different NGSTAR+ NGMAST	203	>15	1471	207
NG_AMS0206	NG_AMS0555	P36	P69	Between-host		1583	1583	15589	16001	3310	NEW2	same MLST, different NGSTAR+ NGMAST	193	>15	1563	216
NG_AMS0206	NG_AMS0480	P36	P39	Between-host		1583	1583	15589	15589	3310	1340	same MLST+ NGMAST, different	120	>15	822	97
NG_AMS0206	NG_AMS0475	P36	P36	Within-host	t1t2	1583	1583	15589	15589	3310	3310	same MLST+	1	<15	4	1
NG_AMS0220	NG_AMS0528	P45	P56	Between-host		7827	7827	10386	UNKN04	38	175	same MLST, different NGSTAR+ NGMAST	51	>15	270	27
NG_AMS0220	NG_AMS0223	P45	P45	Within-host	t1t2	7827	7827	10386	10386	38	38	same MLST+	1	<15	1	0
NG_AMS0220	NG_AMS0405	P45	P10	Between-host		7827	7827	10386	10386	38	38	same MLST+	22	>15	61	18

NG_AMS0221	NG_AMS0575	P44	P77	Between-host		10314	10314	19188	12547	1387	1387	same MLST+ NGSTAR, different NGMAST	37	>15	324	31
NG_AMS0221	NG_AMS0520	P44	P53	Between-host		10314	10314	19188	19188	1387	1387	same MLST+	0	<15	7	1
NG_AMS0221	NG_AMS0538	P44	P61	Between-host		10314	10314	19188	19188	1387	1387	same MLST+	4	<15	15	1
NG_AMS0221	NG_AMS0545	P44	P64	Between-host		10314	10314	19188	19188	1387	1387	same MLST+	3	<15	22	1
NG_AMS0221	NG_AMS0500	P44	P44	Within-host	t1t2	10314	10314	19188	19188	1387	1387	same MLST+	0	<15	5	1
NG_AMS0245	NG_AMS0542	P54	P63	Between-host		1599	1599	13484	11461	2152	520	same MLST, different NGSTAR+ NGMAST	116	>15	750	57
NG_AMS0245	NG_AMS0522	P54	P54	Within-host		1599	1599	13484	13484	2152	2152	same MLST+	1	<15	8	2
NG_AMS0382	NG_AMS0517	P01	P52	Between-host		10314	7822	12547	12547	1387	1387	different MLST, same NGMAST+ NGSTAR	124	>15	741	123
NG_AMS0382	NG_AMS0420	P01	P14	Between-host		10314	10314	12547	16065	1387	1615	same MLST, different NGSTAR+ NGMAST	58	>15	463	32
NG_AMS0382	NG_AMS0407	P01	P10	Between-host		10314	10314	12547	16020	1387	UNKN02	same MLST, different NGSTAR+ NGMAST	72	>15	578	45
NG_AMS0382	NG_AMS0469	P01	P34	Between-host		10314	10314	12547	19178	1387	2730	same MLST, different NGSTAR+ NGMAST	82	>15	633	49
NG_AMS0382	NG_AMS0520	P01	P53	Between-host		10314	10314	12547	19188	1387	1387	same MLST+ NGSTAR, different NGMAST	25	>15	158	16
NG_AMS0382	NG_AMS0500	P01	P44	Between-host		10314	10314	12547	19188	1387	1387	same MLST+ NGSTAR, different NGMAST	25	>15	164	17
NG_AMS0382	NG_AMS0538	P01	P61	Between-host		10314	10314	12547	19188	1387	1387	same MLST+ NGSTAR, different NGMAST	25	>15	166	17
NG_AMS0382	NG_AMS0545	P01	P64	Between-host		10314	10314	12547	19188	1387	1387	same MLST+ NGSTAR, different NGMAST	25	>15	176	17
NG_AMS0382	NG_AMS0386	P01	P01	Within-host	t1t2	10314	10314	12547	12547	1387	1387	same MLST+	0	<15	7	0
NG_AMS0382	NG_AMS0575	P01	P77	Between-host		10314	10314	12547	12547	1387	1387	same MLST+	13	<15	185	17
NG_AMS0383	NG_AMS0384	P02	P02	Within-host		8122	8122	292	292	299	299	same MLST+	0	<15	20	1
NG_AMS0389	NG_AMS0497	P03	P43	Between-host		9363	9363	12302	18797	UNKN01	1039	same MLST, different NGSTAR+ NGMAST	22	>15	105	24
NG_AMS0389	NG_AMS0573	P03	P76	Between-host		9363	9363	12302	NEW6	UNKN01	168	same MLST, different NGSTAR+ NGMAST	43	>15	295	30
NG_AMS0389	NG_AMS0569	P03	P75	Between-host		9363	9363	12302	19275	UNKN01	3194	same MLST, different NGSTAR+ NGMAST	122	>15	1186	110
NG_AMS0389	NG_AMS0511	P03	P49	Between-host		9363	9363	12302	17495	UNKN01	1964	same MLST, different NGSTAR+ NGMAST	132	>15	1721	120
NG_AMS0389	NG_AMS0390	P03	P04	Between-host		9363	9363	12302	17459	UNKN01	301	same MLST, different NGSTAR+ NGMAST	232	>15	2420	191

NG_AMS0389	NG_AMS0442	P03	P24	Between-host		9363	9363	12302	12302	UNKN01	168	same MLST+ NGMAST, different	23	>15	283	18
NG_AMS0389	NG_AMS0437	P03	P22	Between-host		9363	9363	12302	12302	UNKN01	168	same MLST+ NGMAST, different	22	>15	84	20
NG_AMS0389	NG_AMS0408	P03	P11	Between-host		9363	9363	12302	12302	UNKN01	168	same MLST+ NGMAST, different	27	>15	182	22
NG_AMS0389	NG_AMS0515	P03	P51	Between-host		9363	9363	12302	12302	UNKN01	168	same MLST+ NGMAST, different	27	>15	180	22
NG_AMS0389	NG_AMS0400	P03	P08	Between-host		9363	9363	12302	12302	UNKN01	168	same MLST+ NGMAST, different	27	>15	183	23
NG_AMS0389	NG_AMS0396	P03	P03	Within-host	t1t2	9363	9363	12302	12302	UNKN01	UNKN01	same MLST+	0	<15	7	1
NG_AMS0390	NG_AMS0442	P04	P24	Between-host		9363	9363	17459	12302	301	168	same MLST, different NGSTAR+ NGMAST	231	>15	2183	185
NG_AMS0390	NG_AMS0408	P04	P11	Between-host		9363	9363	17459	12302	301	168	same MLST, different NGSTAR+ NGMAST	237	>15	2374	188
NG_AMS0390	NG_AMS0437	P04	P22	Between-host		9363	9363	17459	12302	301	168	same MLST, different NGSTAR+ NGMAST	232	>15	2404	188
NG_AMS0390	NG_AMS0400	P04	P08	Between-host		9363	9363	17459	12302	301	168	same MLST, different NGSTAR+ NGMAST	237	>15	2394	189
NG_AMS0390	NG_AMS0497	P04	P43	Between-host		9363	9363	17459	18797	301	1039	same MLST, different NGSTAR+ NGMAST	231	>15	2424	190
NG_AMS0390	NG_AMS0515	P04	P51	Between-host		9363	9363	17459	12302	301	168	same MLST, different NGSTAR+ NGMAST	236	>15	2382	190
NG_AMS0390	NG_AMS0573	P04	P76	Between-host		9363	9363	17459	NEW6	301	168	same MLST, different NGSTAR+ NGMAST	245	>15	2482	194
NG_AMS0390	NG_AMS0511	P04	P49	Between-host		9363	9363	17459	17495	301	1964	same MLST, different NGSTAR+ NGMAST	255	>15	1861	223
NG_AMS0390	NG_AMS0569	P04	P75	Between-host		9363	9363	17459	19275	301	3194	same MLST, different NGSTAR+ NGMAST	294	>15	2837	255
NG_AMS0390	NG_AMS0391	P04	P04	Within-host		9363	9363	17459	17459	301	301	same MLST+	0	<15	9	1
NG_AMS0392	NG_AMS0577	P05	P78	Between-host		8156	8156	5441	UNKN07	442	442	same MLST+ NGSTAR, different NGMAST	18	>15	167	17
NG_AMS0392	NG_AMS0446	P05	P27	Between-host		8156	8156	5441	UNKN02	442	442	same MLST+ NGSTAR, different NGMAST	53	>15	465	61
NG_AMS0392	NG_AMS0582	P05	P80	Between-host		8156	8156	5441	3674	442	442	same MLST+ NGSTAR, different NGMAST	114	>15	1390	101
NG_AMS0392	NG_AMS0551	P05	P67	Between-host		8156	8156	5441	16928	442	442	same MLST+ NGSTAR, different NGMAST	103	>15	780	113

NG_AMS0392	NG_AMS0536	P05	P60	Between-host		8156	8156	5441	16039	442	442	same MLST+ NGSTAR, different NGMAST	173	>15	1392	131
NG_AMS0392	NG_AMS0394	P05	P06	Between-host		8156	8156	5441	5441	442	442	same MLST+	18	>15	170	15
NG_AMS0392	NG_AMS0503	P05	P46	Between-host		8156	8156	5441	5441	442	442	same MLST+	26	>15	169	19
NG_AMS0392	NG_AMS0393	P05	P05	Within-host		8156	8156	5441	5441	442	442	same MLST+	0	<15	9	1
NG_AMS0392	NG_AMS0492	P05	P42	Between-host		8156	8156	5441	5441	442	442	same MLST+	65	>15	523	41
NG_AMS0392	NG_AMS0502	P05	P45	Between-host		8156	8156	5441	5441	442	442	same MLST+	47	>15	361	53
NG_AMS0392	NG_AMS0454	P05	P28	Between-host		8156	8156	5441	5441	442	442	same MLST+	78	>15	569	73
NG_AMS0394	NG_AMS0577	P06	P78	Between-host		8156	8156	5441	UNKN07	442	442	same MLST+ NGSTAR, different NGMAST	1	<15	21	3
NG_AMS0394	NG_AMS0446	P06	P27	Between-host		8156	8156	5441	UNKN02	442	442	same MLST+ NGSTAR, different NGMAST	56	>15	372	63
NG_AMS0394	NG_AMS0582	P06	P80	Between-host		8156	8156	5441	3674	442	442	same MLST+ NGSTAR, different NGMAST	114	>15	1334	104
NG_AMS0394	NG_AMS0551	P06	P67	Between-host		8156	8156	5441	16928	442	442	same MLST+ NGSTAR, different NGMAST	107	>15	773	116
NG_AMS0394	NG_AMS0536	P06	P60	Between-host		8156	8156	5441	16039	442	442	same MLST+ NGSTAR, different NGMAST	178	>15	1311	137
NG_AMS0394	NG_AMS0503	P06	P46	Between-host		8156	8156	5441	5441	442	442	same MLST+	10	<15	28	8
NG_AMS0394	NG_AMS0395	P06	P06	Within-host		8156	8156	5441	5441	442	442	same MLST+	0	<15	9	0
NG_AMS0394	NG_AMS0492	P06	P42	Between-host		8156	8156	5441	5441	442	442	same MLST+	63	>15	464	37
NG_AMS0394	NG_AMS0502	P06	P45	Between-host		8156	8156	5441	5441	442	442	same MLST+	46	>15	265	50
NG_AMS0394	NG_AMS0454	P06	P28	Between-host		8156	8156	5441	5441	442	442	same MLST+	79	>15	532	77
NG_AMS0397	NG_AMS0398	P07	P07	Within-host		12093	12093	5964	5964	139	139	same MLST+	0	<15	20	7
NG_AMS0400	NG_AMS0497	P08	P43	Between-host		9363	9363	12302	18797	168	1039	same MLST, different NGSTAR+ NGMAST	16	>15	137	20
NG_AMS0400	NG_AMS0569	P08	P75	Between-host		9363	9363	12302	19275	168	3194	same MLST, different NGSTAR+ NGMAST	121	>15	1224	107
NG_AMS0400	NG_AMS0511	P08	P49	Between-host		9363	9363	12302	17495	168	1964	same MLST, different NGSTAR+ NGMAST	135	>15	1726	117
NG_AMS0400	NG_AMS0573	P08	P76	Between-host		9363	9363	12302	NEW6	168	168	same MLST+ NGSTAR, different NGMAST	35	>15	329	25
NG_AMS0400	NG_AMS0408	P08	P11	Between-host		9363	9363	12302	12302	168	168	same MLST+	1	<15	13	0
NG_AMS0400	NG_AMS0515	P08	P51	Between-host		9363	9363	12302	12302	168	168	same MLST+	4	<15	10	3
NG_AMS0400	NG_AMS0437	P08	P22	Between-host		9363	9363	12302	12302	168	168	same MLST+	16	>15	121	16
NG_AMS0400	NG_AMS0442	P08	P24	Between-host		9363	9363	12302	12302	168	168	same MLST+	18	>15	323	16
NG_AMS0400	NG_AMS0403	P08	P08	Within-host	t1t2	9363	9363	12302	12302	168	168	same MLST+	1	<15	7	3
NG_AMS0404	NG_AMS0564	P09	P73	Between-host		7822	7822	14994	19559	1387	1615	same MLST, different NGSTAR+ NGMAST	29	>15	295	24

NG_AMS0404	NG_AMS0430	P09	P20	Between-host		7822	7822	14994	4995	1387	3321	same MLST, different NGSTAR+ NGMAST	700	>15	4414	1463
NG_AMS0404	NG_AMS0444	P09	P26	Between-host		7822	7822	14994	4995	1387	3321	same MLST, different NGSTAR+ NGMAST	699	>15	4477	1477
NG_AMS0404	NG_AMS0517	P09	P52	Between-host		7822	7822	14994	12547	1387	1387	same MLST+ NGSTAR, different NGMAST	145	>15	1505	157
NG_AMS0404	NG_AMS0540	P09	P62	Between-host		7822	7822	14994	14994	1387	1387	same MLST+	28	>15	405	20
NG_AMS0404	NG_AMS0413	P09	P09	Within-host	t1t2	7822	7822	14994	14994	1387	1387	same MLST+	0	<15	10	0
NG_AMS0404	NG_AMS0562	P09	P72	Between-host		7822	7822	14994	14994	1387	1387	same MLST+	55	>15	469	46
NG_AMS0404	NG_AMS0483	P09	P40	Between-host		7822	7822	14994	14994	1387	1387	same MLST+	43	>15	346	49
NG_AMS0404	NG_AMS0553	P09	P68	Between-host		7822	7822	14994	14994	1387	1387	same MLST+	70	>15	571	77
NG_AMS0405	NG_AMS0528	P10	P56	Between-host		7827	7827	10386	UNKN04	38	175	same MLST, different NGSTAR+ NGMAST	39	>15	232	15
NG_AMS0405	NG_AMS0406	P10	P10	Within-host		7827	7827	10386	10386	38	38	same MLST+	0	<15	2	0
NG_AMS0407	NG_AMS0469	P10	P34	Between-host		10314	10314	16020	19178	UNKN02	2730	same MLST, different NGSTAR+ NGMAST	11	<15	73	8
NG_AMS0407	NG_AMS0420	P10	P14	Between-host		10314	10314	16020	16065	UNKN02	1615	same MLST, different NGSTAR+ NGMAST	33	>15	233	24
NG_AMS0407	NG_AMS0520	P10	P53	Between-host		10314	10314	16020	19188	UNKN02	1387	same MLST, different NGSTAR+ NGMAST	93	>15	743	57
NG_AMS0407	NG_AMS0538	P10	P61	Between-host		10314	10314	16020	19188	UNKN02	1387	same MLST, different NGSTAR+ NGMAST	89	>15	717	57
NG_AMS0407	NG_AMS0545	P10	P64	Between-host		10314	10314	16020	19188	UNKN02	1387	same MLST, different NGSTAR+ NGMAST	93	>15	757	57
NG_AMS0407	NG_AMS0575	P10	P77	Between-host		10314	10314	16020	12547	UNKN02	1387	same MLST, different NGSTAR+ NGMAST	77	>15	685	57
NG_AMS0407	NG_AMS0500	P10	P44	Between-host		10314	10314	16020	19188	UNKN02	1387	same MLST, different NGSTAR+ NGMAST	93	>15	750	58
NG_AMS0408	NG_AMS0497	P11	P43	Between-host		9363	9363	12302	18797	168	1039	same MLST, different NGSTAR+ NGMAST	15	<15	135	18
NG_AMS0408	NG_AMS0569	P11	P75	Between-host		9363	9363	12302	19275	168	3194	same MLST, different NGSTAR+ NGMAST	118	>15	1219	106
NG_AMS0408	NG_AMS0511	P11	P49	Between-host		9363	9363	12302	17495	168	1964	same MLST, different NGSTAR+ NGMAST	134	>15	1695	116
NG_AMS0408	NG_AMS0573	P11	P76	Between-host		9363	9363	12302	NEW6	168	168	same MLST+ NGSTAR, different NGMAST	34	>15	325	24
NG_AMS0408	NG_AMS0515	P11	P51	Between-host		9363	9363	12302	12302	168	168	same MLST+	3	<15	15	2
NG_AMS0408	NG_AMS0442	P11	P24	Between-host		9363	9363	12302	12302	168	168	same MLST+	17	>15	319	14
NG_AMS0408	NG_AMS0437	P11	P22	Between-host		9363	9363	12302	12302	168	168	same MLST+	15	<15	120	16

NG_AMS0408	NG_AMS0414	P11	P11	Within-host	t1t2	9363	9363	12302	12302	168	168	same MLST+	0	<15	2	0
NG_AMS0411	NG_AMS0487	P12	P41	Between-host		1599	1599	11461	18147	520	3074	same MLST, different NGSTAR+ NGMAST	52	>15	551	23
NG_AMS0411	NG_AMS0522	P12	P54	Between-host		1599	1599	11461	13484	520	2152	same MLST, different NGSTAR+ NGMAST	49	>15	389	25
NG_AMS0411	NG_AMS0441	P12	P25	Between-host		1599	1599	11461	14764	520	520	same MLST+ NGSTAR, different NGMAST	30	>15	277	19
NG_AMS0411	NG_AMS0425	P12	P17	Between-host		1599	1599	11461	11461	520	520	same MLST+	57	>15	253	38
NG_AMS0411	NG_AMS0542	P12	P63	Between-host		1599	1599	11461	11461	520	520	same MLST+	93	>15	486	45
NG_AMS0411	NG_AMS0412	P12	P12	Within-host		1599	1599	11461	11461	520	520	same MLST+	0	<15	13	3
NG_AMS0415	NG_AMS0416	P13	P13	Within-host		NEW1	NEW1	9918	9918	436	436	same MLST+	0	<15	4	1
NG_AMS0420	NG_AMS0469	P14	P34	Between-host		10314	10314	16065	19178	1615	2730	same MLST, different NGSTAR+ NGMAST	44	>15	291	26
NG_AMS0420	NG_AMS0575	P14	P77	Between-host		10314	10314	16065	12547	1615	1387	same MLST, different NGSTAR+ NGMAST	66	>15	579	42
NG_AMS0420	NG_AMS0520	P14	P53	Between-host		10314	10314	16065	19188	1615	1387	same MLST, different NGSTAR+ NGMAST	81	>15	625	43
NG_AMS0420	NG_AMS0500	P14	P44	Between-host		10314	10314	16065	19188	1615	1387	same MLST, different NGSTAR+ NGMAST	81	>15	627	44
NG_AMS0420	NG_AMS0538	P14	P61	Between-host		10314	10314	16065	19188	1615	1387	same MLST, different NGSTAR+ NGMAST	76	>15	600	44
NG_AMS0420	NG_AMS0545	P14	P64	Between-host		10314	10314	16065	19188	1615	1387	same MLST, different NGSTAR+ NGMAST	80	>15	644	44
NG_AMS0421	NG_AMS0422	P15	P15	Within-host	t1t2	8145	8145	13470	13470	3651	3651	same MLST+	0	<15	18	2
NG_AMS0423	NG_AMS0525	P16	P55	Between-host		11422	11422	UNKN01	3935	2953	193	same MLST, different NGSTAR+ NGMAST	33	>15	325	14
NG_AMS0423	NG_AMS0424	P16	P16	Within-host		11422	11422	UNKN01	UNKN01	2953	2953	same MLST+	0	<15	3	0
NG_AMS0425	NG_AMS0522	P17	P54	Between-host		1599	1599	11461	13484	520	2152	same MLST, different NGSTAR+ NGMAST	87	>15	521	46
NG_AMS0425	NG_AMS0487	P17	P41	Between-host		1599	1599	11461	18147	520	3074	same MLST, different NGSTAR+ NGMAST	101	>15	696	57
NG_AMS0425	NG_AMS0441	P17	P25	Between-host		1599	1599	11461	14764	520	520	same MLST+ NGSTAR, different NGMAST	79	>15	436	55
NG_AMS0425	NG_AMS0542	P17	P63	Between-host		1599	1599	11461	11461	520	520	same MLST+	53	>15	282	29
NG_AMS0425	NG_AMS0428	P17	P17	Within-host	t1t2	1599	1599	11461	11461	520	520	same MLST+	0	<15	4	1
NG_AMS0426	NG_AMS0427	P18	P18	Within-host		8143	8143	5624	5624	426	426	same MLST+	0	<15	4	2
NG_AMS0429	NG_AMS0455	P19	P28	Between-host		11428	11428	NEW8	2992	63	63	same MLST+ NGSTAR, different NGMAST	165	>15	1547	197
NG_AMS0429	NG_AMS0431	P19	P19	Within-host	t1t2	11428	11428	NEW8	NEW8	63	63	same MLST+	1	<15	4	0

NG_AMS0430	NG_AMS0564	P20	P73	Between-host		7822	7822	4995	19559	3321	1615	same MLST, different NGSTAR+ NGMAST	701	>15	4426	1439
NG_AMS0430	NG_AMS0553	P20	P68	Between-host		7822	7822	4995	14994	3321	1387	same MLST, different NGSTAR+ NGMAST	697	>15	4461	1442
NG_AMS0430	NG_AMS0517	P20	P52	Between-host		7822	7822	4995	12547	3321	1387	same MLST, different NGSTAR+ NGMAST	698	>15	4792	1445
NG_AMS0430	NG_AMS0562	P20	P72	Between-host		7822	7822	4995	14994	3321	1387	same MLST, different NGSTAR+ NGMAST	693	>15	4346	1451
NG_AMS0430	NG_AMS0540	P20	P62	Between-host		7822	7822	4995	14994	3321	1387	same MLST, different NGSTAR+ NGMAST	698	>15	4562	1455
NG_AMS0430	NG_AMS0483	P20	P40	Between-host		7822	7822	4995	14994	3321	1387	same MLST, different NGSTAR+ NGMAST	693	>15	4413	1461
NG_AMS0430	NG_AMS0444	P20	P26	Between-host		7822	7822	4995	4995	3321	3321	same MLST+	13	<15	5	4
NG_AMS0430	NG_AMS0432	P20	P20	Within-host	t1t2	7822	7822	4995	4995	3321	3321	same MLST+	0	<15	6	0
NG_AMS0436	NG_AMS0477	P22	P37	Between-host		13734	13734	13489	13489	3171	3171	same MLST+	1	<15	13	2
NG_AMS0437	NG_AMS0497	P22	P43	Between-host		9363	9363	12302	18797	168	1039	same MLST, different NGSTAR+ NGMAST	5	<15	28	11
NG_AMS0437	NG_AMS0569	P22	P75	Between-host		9363	9363	12302	19275	168	3194	same MLST, different NGSTAR+ NGMAST	111	>15	1132	106
NG_AMS0437	NG_AMS0511	P22	P49	Between-host		9363	9363	12302	17495	168	1964	same MLST, different NGSTAR+ NGMAST	128	>15	1658	112
NG_AMS0437	NG_AMS0573	P22	P76	Between-host		9363	9363	12302	NEW6	168	168	same MLST+ NGSTAR, different NGMAST	30	>15	232	23
NG_AMS0437	NG_AMS0442	P22	P24	Between-host		9363	9363	12302	12302	168	168	same MLST+	9	<15	222	13
NG_AMS0437	NG_AMS0515	P22	P51	Between-host		9363	9363	12302	12302	168	168	same MLST+	14	<15	118	17
NG_AMS0437	NG_AMS0438	P22	P22	Within-host	t1t2	9363	9363	12302	12302	168	168	same MLST+	1	<15	4	1
NG_AMS0439	NG_AMS0440	P23	P23	Within-host	t1t2	1580	1580	1195	1195	182	182	same MLST+	1	<15	13	1
NG_AMS0441	NG_AMS0487	P25	P41	Between-host		1599	1599	14764	18147	520	3074	same MLST, different NGSTAR+ NGMAST	55	>15	515	29
NG_AMS0441	NG_AMS0522	P25	P54	Between-host		1599	1599	14764	13484	520	2152	same MLST, different NGSTAR+ NGMAST	71	>15	559	41
NG_AMS0441	NG_AMS0542	P25	P63	Between-host		1599	1599	14764	11461	520	520	same MLST+ NGSTAR, different NGMAST	103	>15	575	57
NG_AMS0441	NG_AMS0443	P25	P25	Within-host	t1t2	1599	1599	14764	14764	520	520	same MLST+	0	<15	6	0
NG_AMS0442	NG_AMS0497	P24	P43	Between-host		9363	9363	12302	18797	168	1039	same MLST, different NGSTAR+ NGMAST	10	<15	244	16
NG_AMS0442	NG_AMS0569	P24	P75	Between-host		9363	9363	12302	19275	168	3194	same MLST, different NGSTAR+ NGMAST	113	>15	1091	102

NG_AMS0442	NG_AMS0511	P24	P49	Between-host		9363	9363	12302	17495	168	1964	same MLST, different NGSTAR+ NGMAST	127	>15	1460	113
NG_AMS0442	NG_AMS0573	P24	P76	Between-host		9363	9363	12302	NEW6	168	168	same MLST+ NGSTAR, different NGMAST	30	>15	378	23
NG_AMS0442	NG_AMS0515	P24	P51	Between-host		9363	9363	12302	12302	168	168	same MLST+	16	>15	323	15
NG_AMS0442	NG_AMS0447	P24	P24	Within-host	t1t2	9363	9363	12302	12302	168	168	same MLST+	1	<15	6	2
NG_AMS0444	NG_AMS0564	P26	P73	Between-host		7822	7822	4995	19559	3321	1615	same MLST, different NGSTAR+ NGMAST	701	>15	4493	1453
NG_AMS0444	NG_AMS0553	P26	P68	Between-host		7822	7822	4995	14994	3321	1387	same MLST, different NGSTAR+ NGMAST	697	>15	4513	1456
NG_AMS0444	NG_AMS0517	P26	P52	Between-host		7822	7822	4995	12547	3321	1387	same MLST, different NGSTAR+ NGMAST	697	>15	4831	1459
NG_AMS0444	NG_AMS0562	P26	P72	Between-host		7822	7822	4995	14994	3321	1387	same MLST, different NGSTAR+ NGMAST	694	>15	4393	1465
NG_AMS0444	NG_AMS0540	P26	P62	Between-host		7822	7822	4995	14994	3321	1387	same MLST, different NGSTAR+ NGMAST	698	>15	4610	1469
NG_AMS0444	NG_AMS0483	P26	P40	Between-host		7822	7822	4995	14994	3321	1387	same MLST, different NGSTAR+ NGMAST	692	>15	4464	1474
NG_AMS0444	NG_AMS0445	P26	P26	Within-host		7822	7822	4995	4995	3321	3321	same MLST+	1	<15	5	1
NG_AMS0446	NG_AMS0503	P27	P46	Between-host		8156	8156	UNKN02	5441	442	442	same MLST+ NGSTAR, different NGMAST	64	>15	374	66
NG_AMS0446	NG_AMS0577	P27	P78	Between-host		8156	8156	UNKN02	UNKN07	442	442	same MLST+ NGSTAR, different NGMAST	56	>15	370	66
NG_AMS0446	NG_AMS0454	P27	P28	Between-host		8156	8156	UNKN02	5441	442	442	same MLST+ NGSTAR, different NGMAST	84	>15	659	80
NG_AMS0446	NG_AMS0492	P27	P42	Between-host		8156	8156	UNKN02	5441	442	442	same MLST+ NGSTAR, different NGMAST	105	>15	782	88
NG_AMS0446	NG_AMS0551	P27	P67	Between-host		8156	8156	UNKN02	16928	442	442	same MLST+ NGSTAR, different NGMAST	77	>15	759	93
NG_AMS0446	NG_AMS0502	P27	P45	Between-host		8156	8156	UNKN02	5441	442	442	same MLST+ NGSTAR, different NGMAST	83	>15	564	96
NG_AMS0446	NG_AMS0582	P27	P80	Between-host		8156	8156	UNKN02	3674	442	442	same MLST+ NGSTAR, different NGMAST	150	>15	1606	139
NG_AMS0446	NG_AMS0536	P27	P60	Between-host		8156	8156	UNKN02	16039	442	442	same MLST+ NGSTAR, different NGMAST	181	>15	1414	150
NG_AMS0446	NG_AMS0451	P27	P27	Within-host	t1t2	8156	8156	UNKN02	UNKN02	442	442	same MLST+	0	<15	8	3
NG_AMS0454	NG_AMS0577	P28	P78	Between-host		8156	8156	5441	UNKN07	442	442	same MLST+ NGSTAR, different NGMAST	78	>15	523	79

NG_AMS0454	NG_AMS0582	P28	P80	Between-host		8156	8156	5441	3674	442	442	same MLST+ NGSTAR, different NGMAST	161	>15	1553	144
NG_AMS0454	NG_AMS0551	P28	P67	Between-host		8156	8156	5441	16928	442	442	same MLST+ NGSTAR, different NGMAST	138	>15	879	146
NG_AMS0454	NG_AMS0536	P28	P60	Between-host		8156	8156	5441	16039	442	442	same MLST+ NGSTAR, different NGMAST	197	>15	1434	160
NG_AMS0454	NG_AMS0503	P28	P46	Between-host		8156	8156	5441	5441	442	442	same MLST+	86	>15	530	81
NG_AMS0454	NG_AMS0492	P28	P42	Between-host		8156	8156	5441	5441	442	442	same MLST+	122	>15	888	97
NG_AMS0454	NG_AMS0502	P28	P45	Between-host		8156	8156	5441	5441	442	442	same MLST+	107	>15	632	108
NG_AMS0455	NG_AMS0530	P28	P57	Between-host		11428	9362	2992	2992	63	63	different MLST, same NGMAST+ NGSTAR	288	>15	1972	315
NG_AMS0456	NG_AMS0458	P29	P29	Within-host	t1t2	12462	12462	5985	5985	42	42	same MLST+	0	<15	5	0
NG_AMS0459	NG_AMS0460	P30	P30	Within-host		13956	13956	13893	13893	1295	1295	same MLST+	0	<15	11	0
NG_AMS0465	NG_AMS0467	P31	P31	Within-host	t1t2	11516	11516	NEW2	NEW2	55	55	same MLST+	0	<15	3	0
NG_AMS0466	NG_AMS0468	P33	P33	Within-host	t1t2	8135	8135	2678	2678	729	729	same MLST+	1	<15	5	1
NG_AMS0472	NG_AMS0474	P35	P35	Within-host	t1t2	11993	11993	387	387	NEW1	NEW1	same MLST+	0	<15	8	1
NG_AMS0477	NG_AMS0482	P37	P37	Within-host	t1t2	13734	13734	13489	13489	3171	3171	same MLST+	1	<15	18	2
NG_AMS0478	NG_AMS0508	P38	P48	Between-host		13489	13489	14700	14700	1225	1225	same MLST+	11	<15	75	12
NG_AMS0478	NG_AMS0485	P38	P38	Within-host	t1t2	13489	13489	14700	14700	1225	1225	same MLST+	0	<15	15	1
NG_AMS0480	NG_AMS0534	P39	P59	Between-host		1583	1583	15589	NEW3	1340	2875	same MLST, different NGSTAR+ NGMAST	113	>15	935	128
NG_AMS0480	NG_AMS0555	P39	P69	Between-host		1583	1583	15589	16001	1340	NEW2	same MLST, different NGSTAR+ NGMAST	127	>15	1016	139
NG_AMS0480	NG_AMS0481	P39	P39	Within-host		1583	1583	15589	15589	1340	1340	same MLST+	2	<15	12	1
NG_AMS0483	NG_AMS0564	P40	P73	Between-host		7822	7822	14994	19559	1387	1615	same MLST, different NGSTAR+ NGMAST	66	>15	571	71
NG_AMS0483	NG_AMS0517	P40	P52	Between-host		7822	7822	14994	12547	1387	1387	same MLST+ NGSTAR, different NGMAST	166	>15	1663	195
NG_AMS0483	NG_AMS0540	P40	P62	Between-host		7822	7822	14994	14994	1387	1387	same MLST+	62	>15	580	72
NG_AMS0483	NG_AMS0562	P40	P72	Between-host		7822	7822	14994	14994	1387	1387	same MLST+	88	>15	649	93
NG_AMS0483	NG_AMS0484	P40	P40	Within-host		7822	7822	14994	14994	1387	1387	same MLST+	0	<15	11	1
NG_AMS0483	NG_AMS0553	P40	P68	Between-host		7822	7822	14994	14994	1387	1387	same MLST+	104	>15	774	124
NG_AMS0487	NG_AMS0522	P41	P54	Between-host		1599	1599	18147	13484	3074	2152	same MLST, different NGSTAR+ NGMAST	90	>15	747	46
NG_AMS0487	NG_AMS0542	P41	P63	Between-host		1599	1599	18147	11461	3074	520	same MLST, different NGSTAR+ NGMAST	119	>15	883	60
NG_AMS0487	NG_AMS0493	P41	P41	Within-host	t1t2	1599	1599	18147	18147	3074	3074	same MLST+	1	<15	8	0
NG_AMS0492	NG_AMS0577	P42	P78	Between-host		8156	8156	5441	UNKN07	442	442	same MLST+ NGSTAR, different NGMAST	62	>15	471	40

NG_AMS0492	NG_AMS0582	P42	P80	Between-host		8156	8156	5441	3674	442	442	same MLST+ NGSTAR, different NGMAST	162	>15	1696	126
NG_AMS0492	NG_AMS0551	P42	P67	Between-host		8156	8156	5441	16928	442	442	same MLST+ NGSTAR, different NGMAST	153	>15	1126	137
NG_AMS0492	NG_AMS0536	P42	P60	Between-host		8156	8156	5441	16039	442	442	same MLST+ NGSTAR, different NGMAST	198	>15	1489	140
NG_AMS0492	NG_AMS0503	P42	P46	Between-host		8156	8156	5441	5441	442	442	same MLST+	70	>15	478	42
NG_AMS0492	NG_AMS0502	P42	P45	Between-host		8156	8156	5441	5441	442	442	same MLST+	93	>15	629	74
NG_AMS0492	NG_AMS0496	P42	P42	Within-host	t1t2	8156	8156	5441	5441	442	442	same MLST+	0	<15	9	0
NG_AMS0497	NG_AMS0515	P43	P51	Between-host		9363	9363	18797	12302	1039	168	same MLST, different NGSTAR+ NGMAST	14	<15	138	19
NG_AMS0497	NG_AMS0573	P43	P76	Between-host		9363	9363	18797	NEW6	1039	168	same MLST, different NGSTAR+ NGMAST	30	>15	254	26
NG_AMS0497	NG_AMS0569	P43	P75	Between-host		9363	9363	18797	19275	1039	3194	same MLST, different NGSTAR+ NGMAST	110	>15	1161	108
NG_AMS0497	NG_AMS0511	P43	P49	Between-host		9363	9363	18797	17495	1039	1964	same MLST, different NGSTAR+ NGMAST	126	>15	1681	117
NG_AMS0497	NG_AMS0498	P43	P43	Within-host		9363	9363	18797	18797	1039	1039	same MLST+	1	<15	4	1
NG_AMS0502	NG_AMS0577	P45	P78	Between-host		8156	8156	5441	UNKN07	442	442	same MLST+ NGSTAR, different NGMAST	46	>15	269	51
NG_AMS0502	NG_AMS0582	P45	P80	Between-host		8156	8156	5441	3674	442	442	same MLST+ NGSTAR, different NGMAST	134	>15	1442	130
NG_AMS0502	NG_AMS0551	P45	P67	Between-host		8156	8156	5441	16928	442	442	same MLST+ NGSTAR, different NGMAST	128	>15	912	142
NG_AMS0502	NG_AMS0536	P45	P60	Between-host		8156	8156	5441	16039	442	442	same MLST+ NGSTAR, different NGMAST	196	>15	1367	161
NG_AMS0502	NG_AMS0503	P45	P46	Between-host		8156	8156	5441	5441	442	442	same MLST+	56	>15	264	54
NG_AMS0503	NG_AMS0577	P46	P78	Between-host		8156	8156	5441	UNKN07	442	442	same MLST+ NGSTAR, different NGMAST	11	<15	34	10
NG_AMS0503	NG_AMS0582	P46	P80	Between-host		8156	8156	5441	3674	442	442	same MLST+ NGSTAR, different NGMAST	122	>15	1323	107
NG_AMS0503	NG_AMS0551	P46	P67	Between-host		8156	8156	5441	16928	442	442	same MLST+ NGSTAR, different NGMAST	115	>15	766	120
NG_AMS0503	NG_AMS0536	P46	P60	Between-host		8156	8156	5441	16039	442	442	same MLST+ NGSTAR, different NGMAST	183	>15	1313	140
NG_AMS0503	NG_AMS0505	P46	P46	Within-host	t1t2	8156	8156	5441	5441	442	442	same MLST+	0	<15	4	0
NG_AMS0506	NG_AMS0510	P47	P47	Within-host	t1t2	14399	14399	19665	19665	UNKN04	UNKN04	same MLST+	2	<15	6	2
NG_AMS0508	NG_AMS0509	P48	P48	Within-host		13489	13489	14700	14700	1225	1225	same MLST+	0	<15	10	1

NG_AMS0511	NG_AMS0515	P49	P51	Between-host		9363	9363	17495	12302	1964	168	same MLST, different NGSTAR+ NGMAST	133	>15	1688	116
NG_AMS0511	NG_AMS0573	P49	P76	Between-host		9363	9363	17495	NEW6	1964	168	same MLST, different NGSTAR+ NGMAST	145	>15	1790	121
NG_AMS0511	NG_AMS0569	P49	P75	Between-host		9363	9363	17495	19275	1964	3194	same MLST, different NGSTAR+ NGMAST	200	>15	2208	191
NG_AMS0511	NG_AMS0512	P49	P49	Within-host		9363	9363	17495	17495	1964	1964	same MLST+	1	<15	11	3
NG_AMS0513	NG_AMS0514	P50	P50	Within-host		10317	10317	5268	5268	178	178	same MLST+	0	<15	5	0
NG_AMS0515	NG_AMS0569	P51	P75	Between-host		9363	9363	12302	19275	168	3194	same MLST, different NGSTAR+ NGMAST	119	>15	1230	106
NG_AMS0515	NG_AMS0573	P51	P76	Between-host		9363	9363	12302	NEW6	168	168	same MLST+ NGSTAR, different NGMAST	37	>15	327	26
NG_AMS0516	NG_AMS0519	P51	P51	Within-host	t1t2	9363	9363	12302	12302	168	168	same MLST+	4	<15	6	2
NG_AMS0517	NG_AMS0575	P52	P77	Between-host		7822	10314	12547	12547	1387	1387	different MLST, same NGMAST+ NGSTAR	127	>15	814	133
NG_AMS0517	NG_AMS0564	P52	P73	Between-host		7822	7822	12547	19559	1387	1615	same MLST, different NGSTAR+ NGMAST	161	>15	1662	175
NG_AMS0517	NG_AMS0540	P52	P62	Between-host		7822	7822	12547	14994	1387	1387	same MLST+ NGSTAR, different NGMAST	147	>15	1676	161
NG_AMS0517	NG_AMS0562	P52	P72	Between-host		7822	7822	12547	14994	1387	1387	same MLST+ NGSTAR, different NGMAST	174	>15	1637	204
NG_AMS0517	NG_AMS0553	P52	P68	Between-host		7822	7822	12547	14994	1387	1387	same MLST+ NGSTAR, different NGMAST	186	>15	1672	222
NG_AMS0517	NG_AMS0518	P52	P52	Within-host		7822	7822	12547	12547	1387	1387	same MLST+	0	<15	14	1
NG_AMS0520	NG_AMS0575	P53	P77	Between-host		10314	10314	19188	12547	1387	1387	same MLST+ NGSTAR, different NGMAST	37	>15	315	29
NG_AMS0520	NG_AMS0545	P53	P64	Between-host		10314	10314	19188	19188	1387	1387	same MLST+	3	<15	14	0
NG_AMS0520	NG_AMS0538	P53	P61	Between-host		10314	10314	19188	19188	1387	1387	same MLST+	4	<15	17	1
NG_AMS0520	NG_AMS0521	P53	P53	Within-host		10314	10314	19188	19188	1387	1387	same MLST+	1	<15	4	0
NG_AMS0525	NG_AMS0526	P55	P55	Within-host		11422	11422	3935	3935	193	193	same MLST+	0	<15	3	1
NG_AMS0528	NG_AMS0529	P56	P56	Within-host	t1t2	7827	7827	UNKN04	UNKN04	175	175	same MLST+	1	<15	16	2
NG_AMS0530	NG_AMS0580	P57	P79	Between-host		9362	9362	2992	NEW7	63	1660	same MLST, different NGSTAR+ NGMAST	321	>15	2729	424
NG_AMS0530	NG_AMS0531	P57	P57	Within-host		9362	9362	2992	2992	63	63	same MLST+	1	<15	2	0
NG_AMS0532	NG_AMS0533	P58	P58	Within-host		1893	1893	860	860	1576	1576	same MLST+	0	<15	12	5
NG_AMS0534	NG_AMS0555	P59	P69	Between-host		1583	1583	NEW3	16001	2875	NEW2	same MLST, different NGSTAR+ NGMAST	189	>15	1652	245
NG_AMS0536	NG_AMS0577	P60	P78	Between-host		8156	8156	16039	UNKN07	442	442	same MLST+ NGSTAR, different NGMAST	178	>15	1315	140

NG_AMS0536	NG_AMS0582	P60	P80	Between-host		8156	8156	16039	3674	442	442	same MLST+ NGSTAR, different NGMAST	241	>15	2161	188
NG_AMS0536	NG_AMS0551	P60	P67	Between-host		8156	8156	16039	16928	442	442	same MLST+ NGSTAR, different NGMAST	204	>15	1596	191
NG_AMS0536	NG_AMS0537	P60	P60	Within-host		8156	8156	16039	16039	442	442	same MLST+	0	<15	3	1
NG_AMS0538	NG_AMS0575	P61	P77	Between-host		10314	10314	19188	12547	1387	1387	same MLST+ NGSTAR, different NGMAST	37	>15	302	30
NG_AMS0538	NG_AMS0539	P61	P61	Within-host		10314	10314	19188	NEW4	1387	1387	same MLST+ NGSTAR, different NGMAST	2	<15	145	3
NG_AMS0538	NG_AMS0545	P61	P64	Between-host		10314	10314	19188	19188	1387	1387	same MLST+	0	<15	30	1
NG_AMS0540	NG_AMS0564	P62	P73	Between-host		7822	7822	14994	19559	1387	1615	same MLST, different NGSTAR+ NGMAST	49	>15	566	46
NG_AMS0540	NG_AMS0562	P62	P72	Between-host		7822	7822	14994	14994	1387	1387	same MLST+	71	>15	623	68
NG_AMS0540	NG_AMS0553	P62	P68	Between-host		7822	7822	14994	14994	1387	1387	same MLST+	83	>15	783	94
NG_AMS0540	NG_AMS0541	P62	P62	Within-host		7822	7822	14994	14994	1387	1387	same MLST+	0	<15	9	1
NG_AMS0542	NG_AMS0543	P63	P63	Within-host		1599	1599	11461	11461	520	520	same MLST+	0	<15	3	2
NG_AMS0545	NG_AMS0575	P64	P77	Between-host		10314	10314	19188	12547	1387	1387	same MLST+ NGSTAR, different NGMAST	37	>15	328	29
NG_AMS0547	NG_AMS0548	P65	P65	Within-host		1901	1901	NEW5	NEW5	128	128	same MLST+	1	<15	9	3
NG_AMS0549	NG_AMS0560	P66	P71	Between-host		11706	11706	17972	UNKN06	1869	1869	same MLST+ NGSTAR, different NGMAST	35	>15	181	38
NG_AMS0549	NG_AMS0576	P66	P77	Between-host		11706	11706	17972	NEW9	1869	1869	same MLST+ NGSTAR, different NGMAST	36	>15	188	39
NG_AMS0549	NG_AMS0557	P66	P70	Between-host		11706	11706	17972	17972	1869	1869	same MLST+	16	>15	64	5
NG_AMS0549	NG_AMS0567	P66	P74	Between-host		11706	11706	17972	17972	1869	1869	same MLST+	71	>15	424	66
NG_AMS0549	NG_AMS0550	P66	P66	Within-host		11706	11706	17972	17972	1869	1869	same MLST+	15	<15	67	6
NG_AMS0551	NG_AMS0577	P67	P78	Between-host		8156	8156	16928	UNKN07	442	442	same MLST+ NGSTAR, different NGMAST	107	>15	747	119
NG_AMS0551	NG_AMS0582	P67	P80	Between-host		8156	8156	16928	3674	442	442	same MLST+ NGSTAR, different NGMAST	185	>15	1808	174
NG_AMS0551	NG_AMS0552	P67	P67	Within-host		8156	8156	16928	16928	442	442	same MLST+	0	<15	12	2
NG_AMS0553	NG_AMS0564	P68	P73	Between-host		7822	7822	14994	19559	1387	1615	same MLST, different NGSTAR+ NGMAST	89	>15	733	99
NG_AMS0553	NG_AMS0562	P68	P72	Between-host		7822	7822	14994	14994	1387	1387	same MLST+	95	>15	676	103
NG_AMS0553	NG_AMS0554	P68	P68	Within-host	t1t2	7822	7822	14994	14994	1387	1387	same MLST+	0	<15	9	0
NG_AMS0555	NG_AMS0556	P69	P69	Within-host		1583	1583	16001	UNKN05	NEW2	NEW2	same MLST+ NGSTAR, different NGMAST	0	<15	5	1
NG_AMS0557	NG_AMS0560	P70	P71	Between-host		11706	11706	17972	UNKN06	1869	1869	same MLST+ NGSTAR, different NGMAST	33	>15	174	33

NG_AMS0557	NG_AMS0576	P70	P77	Between-host		11706	11706	17972	NEW9	1869	1869	same MLST+ NGSTAR, different NGMAST	32	>15	185	33
NG_AMS0557	NG_AMS0567	P70	P74	Between-host		11706	11706	17972	17972	1869	1869	same MLST+	67	>15	426	62
NG_AMS0557	NG_AMS0558	P70	P70	Within-host		11706	11706	17972	17972	1869	1869	same MLST+	2	<15	8	0
NG_AMS0560	NG_AMS0576	P71	P77	Between-host		11706	11706	UNKN06	NEW9	1869	1869	same MLST+ NGSTAR, different NGMAST	5	<15	42	11
NG_AMS0560	NG_AMS0567	P71	P74	Between-host		11706	11706	UNKN06	17972	1869	1869	same MLST+ NGSTAR, different NGMAST	49	>15	320	48
NG_AMS0560	NG_AMS0561	P71	P71	Within-host		11706	11706	UNKN06	17972	1869	1869	same MLST+ NGSTAR, different NGMAST	0	<15	15	0
NG_AMS0562	NG_AMS0564	P72	P73	Between-host		7822	7822	14994	19559	1387	1615	same MLST, different NGSTAR+ NGMAST	79	>15	652	69
NG_AMS0562	NG_AMS0563	P72	P72	Within-host		7822	7822	14994	14994	1387	1387	same MLST+	0	<15	6	1
NG_AMS0564	NG_AMS0565	P73	P73	Within-host		7822	7822	19559	19559	1615	1615	same MLST+	0	<15	11	0
NG_AMS0567	NG_AMS0576	P74	P77	Between-host		11706	11706	17972	NEW9	1869	1869	same MLST+ NGSTAR, different NGMAST	48	>15	312	46
NG_AMS0567	NG_AMS0568	P74	P74	Within-host		11706	11706	17972	17972	1869	1869	same MLST+	0	<15	10	3
NG_AMS0569	NG_AMS0573	P75	P76	Between-host		9363	9363	19275	NEW6	3194	168	same MLST, different NGSTAR+ NGMAST	128	>15	1257	115
NG_AMS0569	NG_AMS0571	P75	P75	Within-host	t1t2	9363	9363	19275	19275	3194	3194	same MLST+	1	<15	6	1
NG_AMS0573	NG_AMS0574	P76	P76	Within-host		9363	9363	NEW6	NEW6	168	168	same MLST+	2	<15	8	2
NG_AMS0577	NG_AMS0582	P78	P80	Between-host		8156	8156	UNKN07	3674	442	442	same MLST+ NGSTAR, different NGMAST	111	>15	1329	106
NG_AMS0577	NG_AMS0578	P78	P78	Within-host		8156	8156	UNKN07	UNKN07	442	442	same MLST+	1	<15	10	0
NG_AMS0580	NG_AMS0581	P79	P79	Within-host		9362	9362	NEW7	NEW7	1660	1660	same MLST+	0	<15	0	0
NG_AMS0582	NG_AMS0583	P80	P80	Within-host		8156	8156	3674	3674	442	442	same MLST+	0	<15	9	0
